# Variants of the 5′-terminal region of p53 mRNA influence the ribosomal scanning and translation efficiency

**DOI:** 10.1038/s41598-018-20010-2

**Published:** 2018-01-24

**Authors:** Paulina Zydowicz-Machtel, Agata Swiatkowska, Łukasz Popenda, Agnieszka Gorska, Jerzy Ciesiołka

**Affiliations:** 1 Institute of Bioorganic Chemistry, Polish Academy of Sciences, Noskowskiego 12/14, 61-704 Poznan, Poland; 2 NanoBioMedical Centre, Adam Mickiewicz University in Poznan, Umultowska 85, 61-614 Poznan, Poland

## Abstract

The p53 protein is one of the major cell cycle regulators. The protein is expressed as at least twelve protein isoforms resulting from the use of alternative promoters, alternative splicing or downstream initiation codons. Importantly, there is growing evidence that translation initiation of p53 mRNA may be regulated by the structure and length of the naturally occurring variants of the 5′-terminal region of p53 mRNA transcripts. Here, several mRNA constructs were synthesized with variable length of the p53 5′-terminal regions and encoding luciferase reporter protein, and their translation was monitored continuously *in situ* in a rabbit reticulocyte lysate system. Moreover, four additional mRNA constructs were prepared. In two constructs, the structural context of AUG1 initiation codon was altered while in the other two constructs, characteristic hairpin motifs present in the p53 5′-terminal region were changed. Translation of the last two constructs was also performed in the presence of the cap analogue to test the function of the 5′-terminal region in cap-independent translation initiation. Superposition of several structural factors connected with the length of the 5′-terminal region, stable elements of the secondary structure, structural environment of the initiation codon and IRES elements greatly influenced the ribosomal scanning and translation efficiency.

## Introduction

The translation process determines the protein composition in the cell, and the translatome changes occurring during cell development or induced by external stimuli. Protein translation is a complex event which enables decoding of genetic information and turning it into a variety of proteins. One of the most essential steps of translation is its initiation, which involves a large number of initiation factors and may be regulated at different levels^1–4^. Identification of the AUG start codon is a crucial stage during the initiation and is preceded by the assembly of 43 S pre-initiation complex (PIC) including the 40 S small ribosomal subunit and initiator Met-tRNA. Once PIC binds to the cap structure it scans the 5′-terminal region in the 3′ direction and an ATP-dependent manner in order to find the initiation codon^5,6^. This ribosomal scanning mechanism is considered to be the predominant model of translation initiation in *Eucaryota*^1,7,8^ in contrast to prokaryotic initiation involving direct rRNA – mRNA base-pairing interaction^9,10^.

The speed of the ribosome movement along the 5′ untranslated region (5′UTR) of mRNA is a vital factor for the protein biosynthesis because it influences the overall translation efficiency. Moreover, the duration of ribosomal scanning is not the same for all mRNAs. Such differences may result from the number and stability of the secondary structure motifs in the 5′UTRs. Highly structured 5′UTRs are unfavourable for 43 S PIC since the complex can only bind to a single stranded region. Therefore, all double-stranded structures must undergo unwinding^11,12^, which causes a delay in ribosomal scanning and a decrease in translation efficiency^13,14^. In the mammalian reconstituted system, eIF4A together with eIF4F, eIF4B, ATP and DEAD-box RNA helicases have been shown to facilitate scanning through structured 5′UTRs^15^. However, recent data exclude the direct role of DHX29 protein in RNA unwinding. It might rather bind the initiation complex, establishing contacts with different subunits of eIF3, thereby causing the rearrangement of the ribosomal complex. The remodelled complex reveals higher processivity in the unwinding of the mRNA secondary structure during scanning^16^. Furthermore, the length of the 5′UTR may also be a limiting factor for the translation efficiency^17^. Reduction of the 5′UTR below 20 nt may result in a decline of translation initiation because of the insufficient length for the PIC binding^2,17^. Another study on model mRNAs shows that during translation initiation the length of the 5′UTR strongly influences the scanning time; besides, it has been demonstrated that this correlation is linear^18^.

Recently, high-throughput sequencing techniques have been used to study the determinants of protein synthesis. The dynamics of ribosomal scanning has been revealed by translation complex profiling in yeast^19^. Multiple mRNAs with unstructured and short 5′UTRs feature smooth distribution of scanning 40 S ribosomal small subunits (SSU) throughout their 5′UTRs. Interestingly, mRNAs with longer 5′UTRs or with short upstream ORFs displayed evidence of a scanning block in which the SSU accumulated. However, in those mRNAs, it remains to be revealed whether the SSU clustering could be attributed to uORFs, RNA structure or binding of specific factors^19^. A computational model of translation in a yeast cell has also been proposed^20^. Moreover, using a large set of synthetic GFP genes the authors have shown experimentally that 5′ mRNA folding plays a predominant role in determining protein levels in *S. cerevisiae*^20^, just like it had earlier been shown in *E. coli*^21^. Although high-throughput approaches have supplied a lot of invaluable information on global protein synthesis in the cell, the data have to be confronted with translation processes of individual mRNAs. An attractive object of such studies is the mRNA encoding p53 protein, in particular, with regard to investigating the role of its 5′ untranslated region in translation initiation.

The p53 protein has been recognized as one of the most important tumour suppressors. In normal cell conditions, p53 protein is maintained at a low level through the human double minute 2 protein (HDM2), which is an E3 ubiquitin ligase, and which targets the p53 for proteasomal degradation^22,23^. However, under different stress conditions in the cell, the p53-HDM2 interaction is inhibited, as a result of which p53 is accumulated and it activates various factors, which have the ability to activate DNA repair mechanisms or promote apoptotic cell death^24,25^. Moreover, it has been demonstrated that HDM2 does not only interact with the p53 protein but it also binds to p53 mRNA and that this interaction stimulates p53 translation^26^. Importantly, the p53 protein is expressed as over 12 protein isoforms resulting from the use of alternative promoters, alternative splicing or downstream initiation codon^24,27,28^. These isoforms are expressed differentially in a tissue-dependent manner and they tend to be regulated under various stress conditions^29,30^. So far, the clinical importance of those isoforms is not fully understood.

There is growing evidence that translation of p53 mRNA may be influenced by the secondary structure of 5′UTRs of various p53 mRNA transcripts naturally occurring in the cell^31–33^. In this regulation, a structured RNA region called IRES (*Internal Ribosome Entry Site*) is also involved. It is located in the 5′UTR and may trigger translation initiation when cap-dependent translation is impaired^34–36^. In addition, the p53 mRNA regulation may be performed by proteins that bind to the 5′UTR of p53 mRNA^31,33^.

Here, we attempted to answer the question how the variable length and structure of 5′UTR influence the ribosome scanning and translation initiation of p53 mRNA. We performed an *in vitro* translation analysis in rabbit reticulocyte lysate, using mRNA constructs with a different length of 5′UTRs and encoding *Renilla* luciferase reporter protein, continuously monitoring protein synthesis *in situ*. Moreover, four additional mRNA constructs were prepared. In two constructs, the structural context of the initiation AUG codon was altered, and in the two other, the characteristic hairpin motifs G56-C169 and U180-A218 present in the 5′UTR of p53 mRNA had been changed. Measurements of the ribosomal scanning time and translation efficiency enabled to determine the effect of the structural alteration of 5′UTR on the translation initiation.

## Results

### Structural alterations within the 5′-terminal region of p53 mRNA influence translation efficiency from both initiation codons AUG1 and AUG2

**Figure 1:**
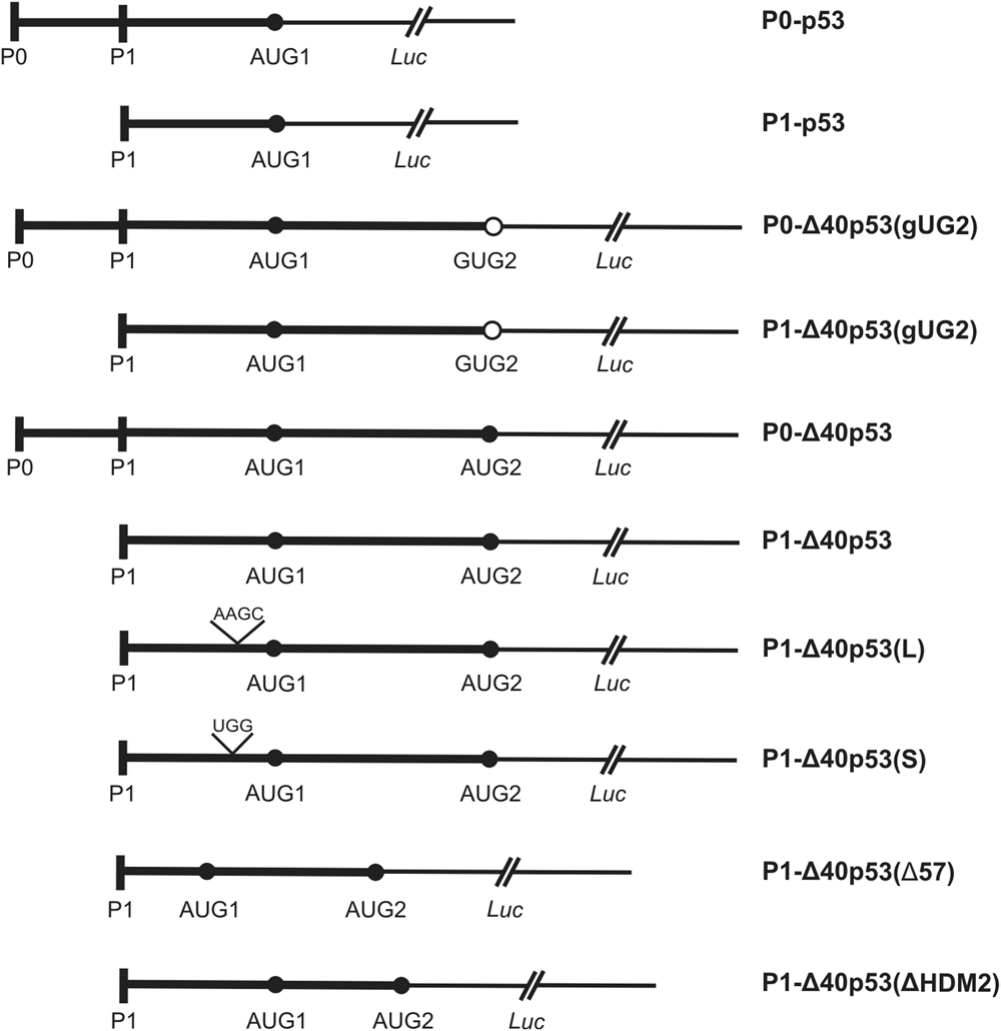
Schematic representation of the RNA constructs consisting of various 5′-terminus of p53 mRNA and the *Renilla* luciferase coding region. P0 and P1 stand for transcription promoter regions while AUG1 and AUG2 indicate translation start codons. The codon AUG2 was mutated to GUG codon for valine in the P1-Δ40p53(gUG2) and P0-Δ40p53(gUG2) constructs. Nucleotide substitutions in the P1-Δ40p53(L) and P0-Δ40p53(S) constructs are marked on the scheme. In the P1-Δ40p53(Δ57) and P1-Δ40p53(ΔHMD2) constructs two hairpin motifs were mutated (see Fig. 5).

In order to establish how structural alterations within the 5′-terminal region of p53 mRNA influence translation several model mRNA constructs were synthesized. The mRNA constructs consisted of various 5′-terminal regions of p53 mRNA and an identical coding sequence of *Renilla* luciferase (Figs 1, 3, 4 and 5, in which the secondary structures of the 5′-terminal regions of the constructs are shown). Synthesis of some constructs has been described in our earlier studies^37^. The 5′ ends of the mRNA constructs corresponded to transcription initiation sites P0 or P1 of the *TP53* gene. The 5′ untranslated regions of p53 mRNA were terminated with initiation codons AUG1 for p53 protein or AUG2 for Δ40p53 isoform. The constructs P0-Δ40p53(gUG2) and P1-Δ40p53(gUG2) contained point mutation in the first position of AUG2 to introduce valine and to block translation initiation from that site. In two other constructs the AUG1 structural context was altered to double-stranded region in P1-Δ40p53(S), or to internal loop in P1-Δ40p53(L). Finally, in the P1-Δ40p53(Δ57) construct the bottom part of the G56-C169 hairpin and the apical loop region were deleted. The nucleotides spanning positions 57–73, 100–125 and 145–168 were removed. In the P1-Δ40p53(ΔHDM2) construct the nucleotides spanning positions 180–215 were deleted. This resulted in the removal of the U180-A218 hairpin, which has earlier been shown to be recognized by HDM2 protein^26^. Structural analyses of new RNA constructs were performed by Pb^2+^-induced cleavage^38,39^ and/or SHAPE^40^ approaches to confirm all the changes introduced within the 5′-terminal region of p53 mRNA (Suppl. Fig. S2a–e).

**Figure 2:**
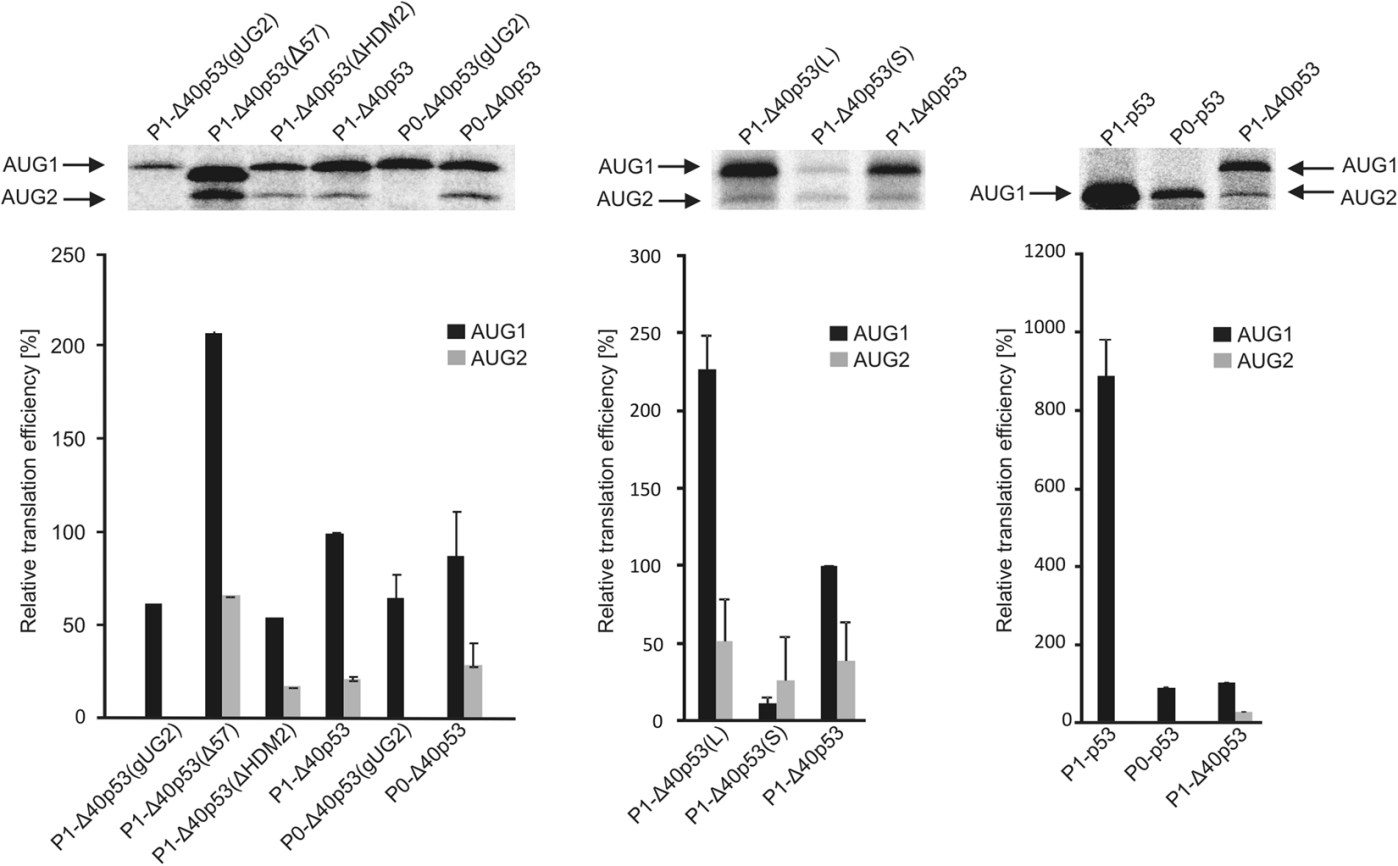
*In vitro* translation of the model mRNA constructs from AUG1 and AUG2 initiation codons performed in the rabbit reticulocyte lysate (RRL) system. The autoradiograms show the translation products and the bar charts quantitatively display the translation efficiency of model mRNAs from two initiation codons. All values are averages of at least three independent experiments and were normalized to the translation efficiency of product synthesized from AUG1 in P1-Δ40p53 mRNA. The full-length autoradiograms were included in Suppl. Fig. S5.

The synthesized mRNA constructs were translated *in vitro* in rabbit reticulocyte lysate (RRL) for 30 min in the presence of radioactive aminoacid ^35^S-methionine (Fig. 2). Additionally, luciferase production from each RNA construct was confirmed by western blot (Suppl. Fig. S3). It turned out that any structural alterations within the 5′ non-coding regions of the constructs caused substantial changes in the translation efficiency from AUG1 or/and AUG2 when compared to translation of the P1-Δ40p53 construct, taken as a reference. The most pronounced effect occurred when AUG1 was placed in a double-stranded region in P1-Δ40p53(S) (Fig. 4a) which resulted in a very strong decrease of translation from AUG1 and smaller reduction of translation efficiency from AUG2. On the other hand, embedding AUG1 in a single-stranded internal loop in P1-Δ40p53(L) (Fig. 4a) caused an approx. 2-fold increase of translation efficiency from AUG1. Interestingly, an improvement of translation from AUG2 by approx. 30% was also observed. The lack of AUG2 in the RNA construct P1-Δ40p53(gUG2) resulted in a slightly lower protein synthesis from AUG1 in contrast to the translation efficiency of its non-mutated counterpart, P1-Δ40p53. An extremely large impact on translation efficiency was observed in the case of P1-Δ40p53(Δ57) in which the G56-C169 hairpin had been shortened. Remarkably, the protein synthesis from both initiation codons, AUG1 and AUG2, increased approximately 2-fold. However, an opposite effect was observed for the P1-Δ40p53(ΔHDM2) construct in which the U180-A218 hairpin had been removed. The translation efficiency from AUG1 decreased by about 50% and slight reduction of protein synthesis from AUG2 was also observed (Fig. 2).

### The impact of various 5′-terminal regions of p53 mRNA on ribosomal scanning and translation efficiency of model mRNA constructs

To further elucidate to what extent the secondary structure of the 5′-terminal regions of p53 mRNA present in model mRNA constructs influences translation initiation we applied a *Renilla* Luciferase Reporter Gene assay developed by the Spirin’s group^18,41^. The assay allows continuous measurement of the newly synthesized reporter protein - luciferase during *in vitro* translation performed in cell lysate systems. Luciferase activity is manifested by conversion of coelenterazine substrate to coelenteramide with light emission which is measured in a spectrophotometer. A kink at the initial rise of the curve corresponds to the moment of appearance of luciferase activity and represents the completion of protein synthesis (i.e. combined duration of the initiation, elongation and termination) indicating the average translation time. The detailed description of the calculation of the full-translation time of first translation rounds values is provided in Supplementary Information. Further in the text we use the term ‘full-translation time’ for simplification. In the case of mRNA constructs with two initiation codons AUG1 and AUG2, fusion luciferase with additional 40 aminoacids attached at its N-terminus was synthesized from AUG1 while the synthesis of the full-length luciferase was initiated from AUG2. Importantly, it has been shown that an engineered *Renilla* luciferase with 11 aminoacids^42^ and firefly luciferase with 23 aminoacids^43^ attached at their N-terminus retained their full enzymatic activity. Therefore, the 40-aminoacid-long tail is likely to have a little impact on the enzymatic activity of fusion luciferase.

Additionally, we found that the determination of maximal values of luminescence activity allows comparing the translation efficiencies of the model mRNAs (see Supplementary Information). These values better represent the relative translation abilities than the amounts of synthesized proteins determined after the specified single time points after translation initiation. However, the disadvantage of the luminescence approach is that it does not allow separation and quantification of the various translation products originating from the same mRNA construct.

#### mRNA constructs with single initiation codon AUG1

**Figure 3:**
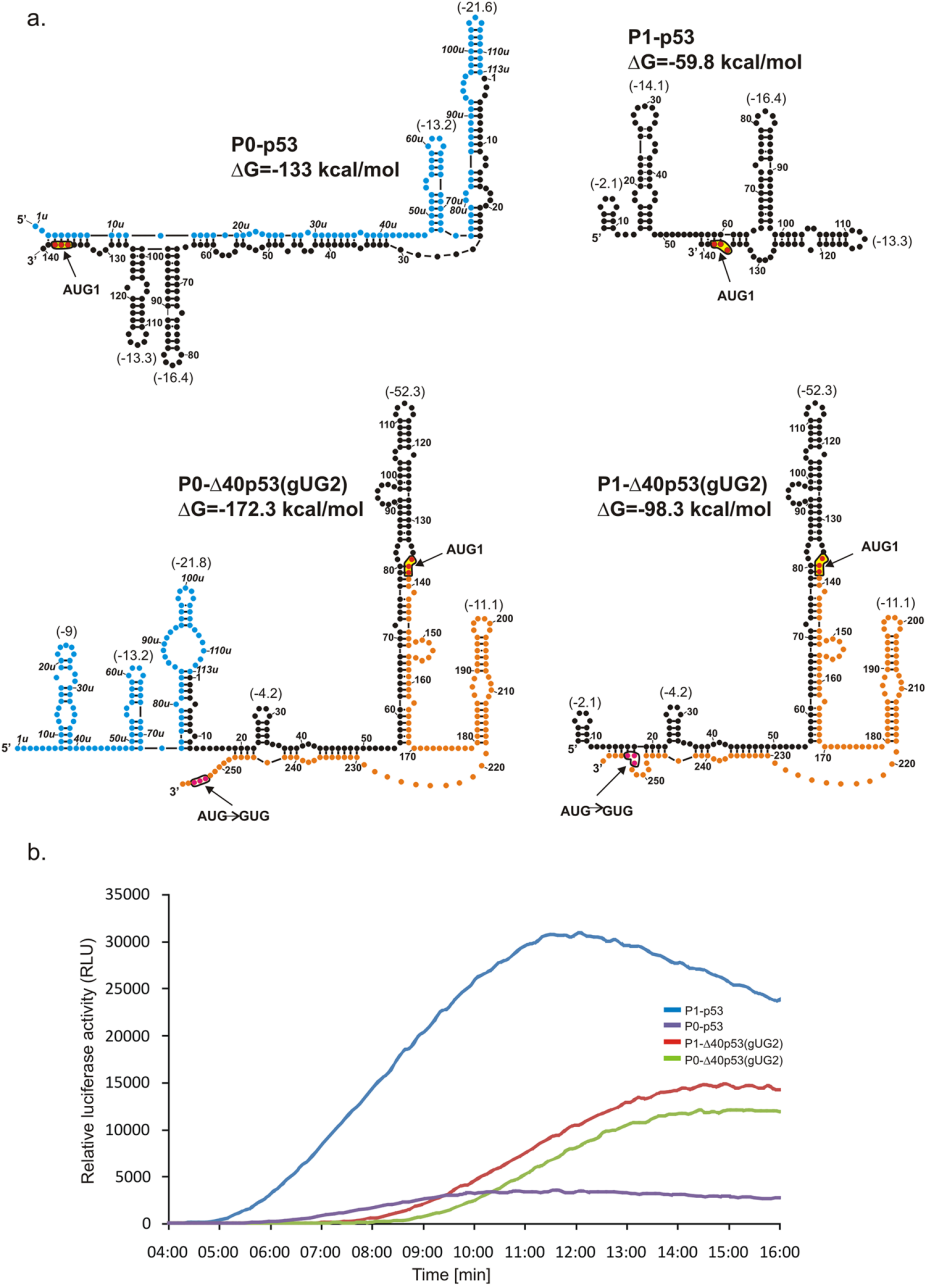
Secondary structure models of the 5′-terminal regions of mRNA constructs with single initiation AUG1 codon and kinetics of their *in vitro* translation followed by a luciferase reporter gene assay. (**a**) Secondary structure models of the 5′UTRs of constructs: P0-p53, P1-p53, P0-Δ40p53(gUG2), and P1-Δ40p53(gUG2). The predicted ΔG value (kcal/mol) for each 5′UTR is indicated and ΔG values of selected structural motifs are also shown in brackets. Colours denote parts of the 5′-terminal region of p53 mRNA: blue – the region between P0 and P1 transcription promoters; black - the 5′ untranslated region downstream P1 promoter; orange - p53 open reading frame (ORF). (**b**) The relative luciferase activity (RLU) was measured during translation of each mRNA construct using a luminometer in 1 s periods with 9-second intervals, for 16 minutes. The data illustrate approximate values of maximal translation efficiency for each construct.

Secondary structures of four mRNA constructs: P0-p53, P1-p53, P0-Δ40p53(gUG2) and P1-Δ40p53(gUG2) are shown in Fig. 3a. Importantly, these constructs contain only one initiation codon AUG1. In the first two constructs this codon immediately precedes the reporter sequence encoding luciferase while in the other two constructs an additional 120-nucleotide-long stretch of p53 sequence is present downstream of AUG1, which ends with a gUG triplet in place of AUG2 codon in p53 mRNA. As a result, these transcripts encode reporter luciferase with additional 40 aminoacids attached at its N-terminus.

Representative data of relative luminescence activity, which increases in time during translation of the mRNA constructs, is shown in Fig. 3b. For the purpose of quantification we used data gathered from three independent experiments and the full-translation time value was calculated for each construct (Table 1). A different length, nucleotide sequence and/or structure of the 5′ non-coding regions of the constructs also results in large differences in the amounts of the synthesized proteins, which are expressed as maximal luminescence activities (Table 1). These values are expressed as relative values to those determined for the P1-Δ40p53 construct, which has the major form of the 5′-terminal region of p53 mRNA occurring in the cell.

**Table 1:** Comparison of full-translation time and maximal luminescence activity for each mRNA construct.

mRNA construct	Full-translation time [min:s]	Maximal luminescence activity [%]
P0-p53	07:08 ± 00:16	33 ± 5
P1-p53	05:46 ± 00:02	300 ± 30
P0-Δ40p53(gUG2)	08:31 ± 00:10	60 ± 12
P1-Δ40p53(gUG2)	08:14 ± 00:09	69 ± 11
P0-Δ40p53	07:35 ± 00:15	33 ± 0.3
P1-Δ40p53	06:58 ± 00:14	100
P1-Δ40p53(L)	07:35 ± 00:08	169 ± 10
P1-Δ40p53(S)	08:00 ± 00:03	90 ± 4
P1-Δ40p53(Δ57)	07:57 ± 00:04	237 ± 9
P1-Δ40p53(ΔHDM2)	08:58 ± 00:04	58 ± 9

Full-translation time with standard deviation was calculated for each construct based on three independent experiments. The maximal luminescence activity obtained for each construct was normalized to the value obtained for the P1-Δ40p53 variant and standard deviations are indicated in the table.

Upon comparison of the two constructs starting from P0 and P1 promoters, with the P0-p53 and P1-p53 non-coding regions, it was observed that the ribosomal full-translation time for the P0-beginning transcript was longer, and an additional 5′ sequence stretch generated a delay in scanning (7:08 min and 5:46 min, respectively). This difference in time corresponds to scanning of additional 113 nucleotides of the 5′ non-coding region in the longer transcript, giving the scanning rate of 1.4 nt/s. For the next two transcripts, P0-Δ40p53(gUG2) and P1-Δ40p53(gUG2), the ribosomal full-translation time amounted to 8:31 min and 8:14 min, respectively. The calculated rate of scanning the same 113 nucleotide-long stretch of 6.6 nt/s is almost 5 times higher than that estimated based on translation of the P0-p53 and P1-p53 constructs. Clearly, both non-coding stretches are scanned at two different rates. This can be explained by their different secondary structure folding. In P0-p53 almost the entire region has a double-stranded character while in P0-Δ40p53(gUG2) it folds into a few hairpins with a relatively low stability.

While comparing the maximal luciferase activity values (Table 1), what is the most striking is the translation efficiency of P1-p53 which is approximately 10-fold higher than that determined for P0-p53. The former construct is shorter and less structured which may explain its higher translation efficiency. The translation efficiencies of P0-Δ40p53(gUG2) and P1-Δ40p53(gUG2) are similar and they amount to 60-70% of the value determined for P1-Δ40p53, as a reference (Table 1).

#### mRNA constructs with two initiation codons AUG1 and AUG2

**Figure 4:**
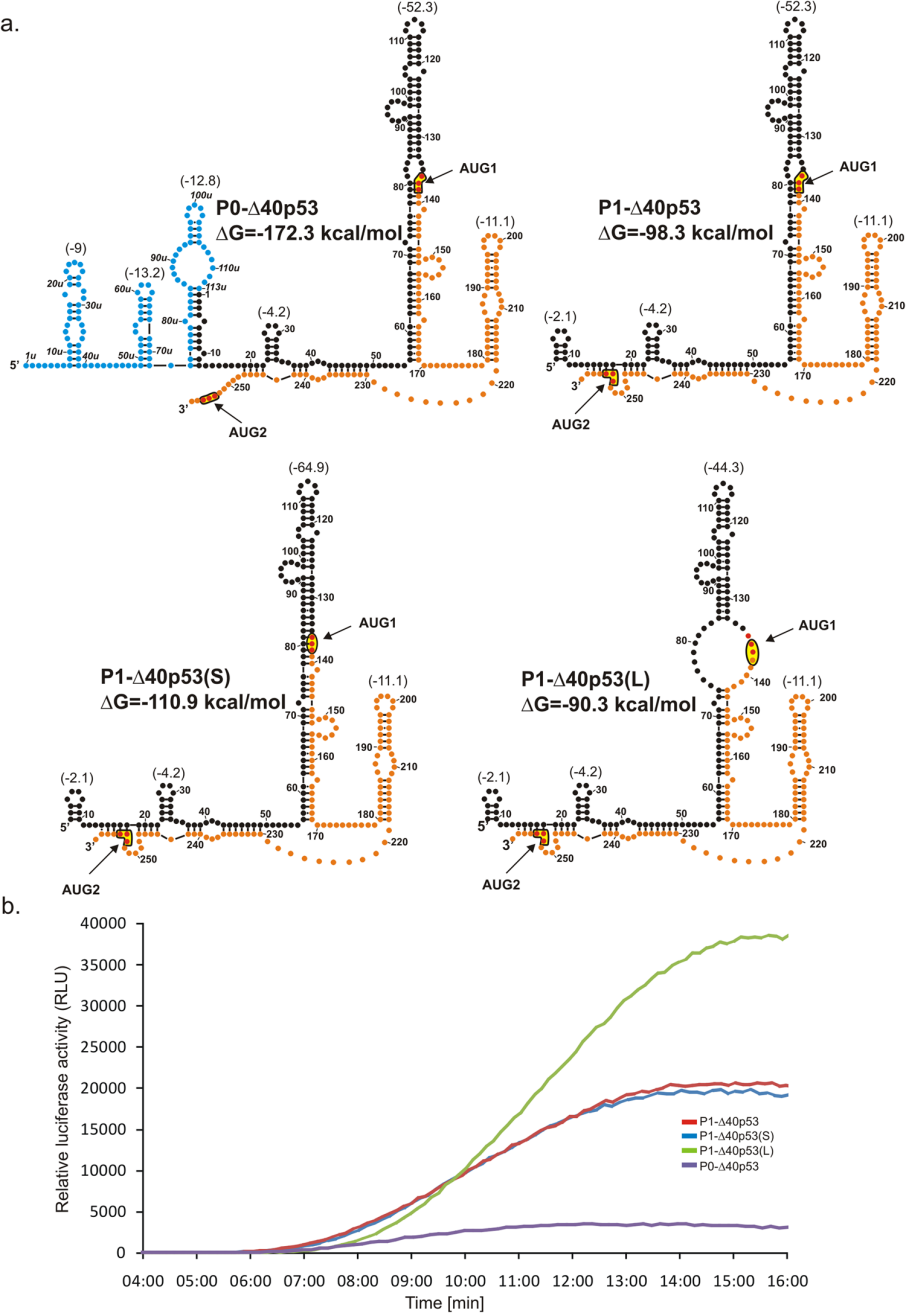
Secondary structure models of 5′-terminal regions of mRNA constructs with two initiation codons AUG1 and AUG2 and kinetics of their *in vitro* translation followed by a luciferase reporter gene assay. (**a**) Secondary structure arrangement of the 5′UTRs of constructs: P0-Δ40p53, P1-Δ40p53, P1-Δ40p53(S) and P1-Δ40p53(L). The predicted ΔG values for each 5′UTR and for selected structural motifs are shown. Colours denote parts of the 5′-terminal region of p53 mRNA as described in the legend to Fig. 2. (**b**) The relative luciferase activity (RLU) was measured during translation of each mRNA construct using a luminometer in 1 s periods with 9-second intervals, for 16 minutes.

Four mRNA constructs: P0-Δ40p53, P1-Δ40p53, P1-Δ40p53(S) and P1-Δ40p53(L) contained two initiation codons AUG1 and AUG2 (Fig. 4a). Therefore, during their translation a mixture of two protein products was synthesized. Based on the data shown in Fig. 2, the ratio of the products initiated from AUG1 and from AUG2 was similar for the studied constructs and was estimated at approx. 3:1 to 4:1. Thus, the major protein that corresponded to the translation initiation from AUG1 had a greater influence on the parameters calculated from the fluorescence assay than the minor product initiated from AUG2.

**Figure 5:**
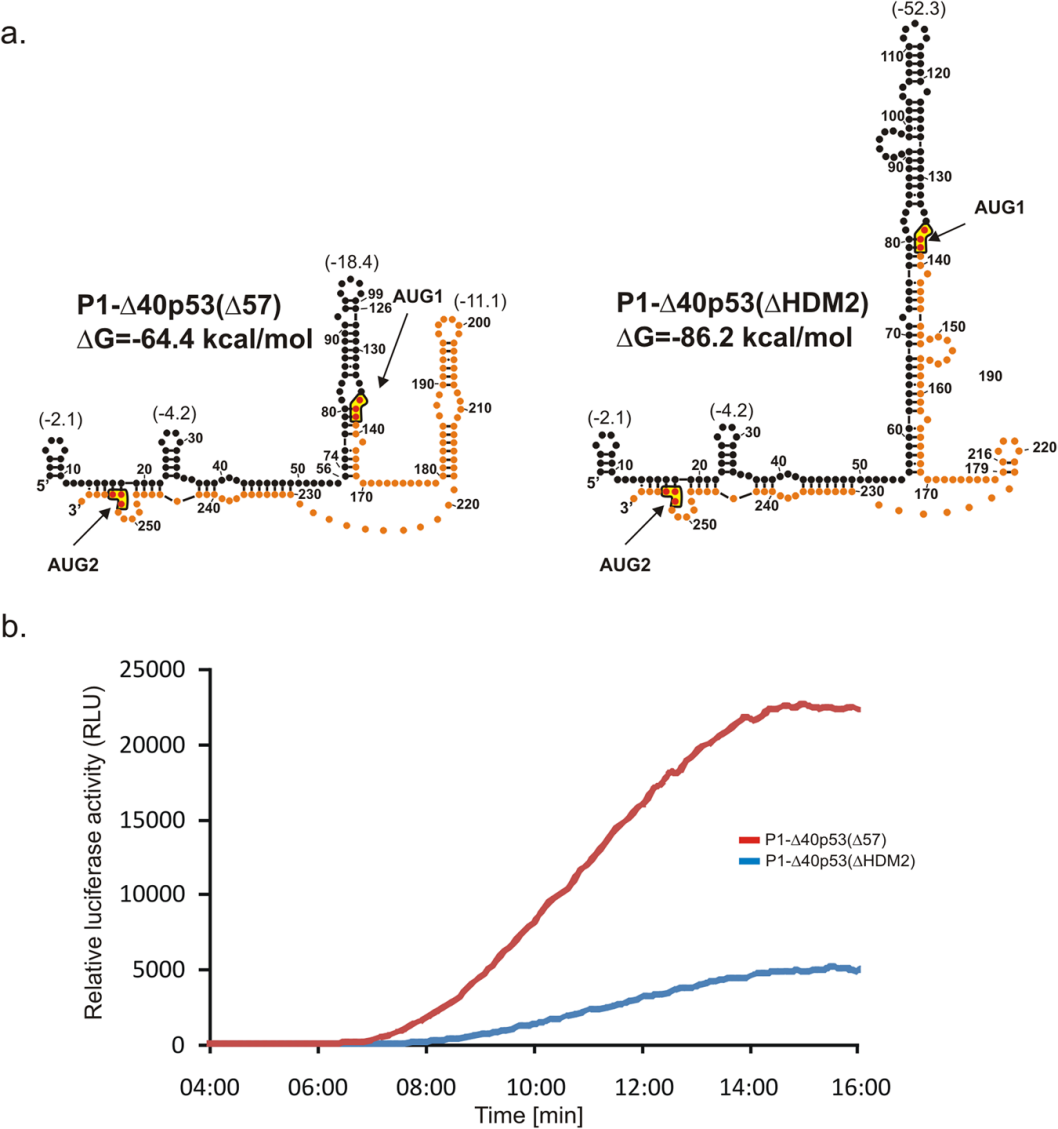
Secondary structure models of 5′-terminal regions of two mRNA constructs in which hairpins G56-C169 and U180-A218 were mutated and kinetics of their *in vitro* translation followed by a luciferase reporter gene assay. (**a**) Secondary structure models of the 5′UTRs of constructs: P1-Δ40p53(Δ57) and P1-Δ40p53(ΔHDM2). The predicted ΔG values for 5′UTR of each construct and for selected structural motifs are indicated. Parts of the 5′-terminal region of p53 mRNA are denoted as in Fig. 3. (**b**) The relative luciferase activity (RLU) was measured during translation of each mRNA construct in 1 s periods with 9-second intervals, for 16 minutes. The data illustrate approximate values of maximal translation efficiency for each construct.

When comparing the two transcripts P0-Δ40p53 and P1-Δ40p53 it turns out that the ribosomal full-translation time for P0-beginning transcript is longer (Table 1), and an additional 5′ sequence stretch generates a delay in scanning (7:35 and 6:58 min, respectively). This delay corresponds to additional 113 nucleotides in the longer transcript, which gives the scanning rate of 3.0 nt/s. The value is half of that determined for the same sequence stretch based on translation of the P0-Δ40p53(gUG2) and P1-Δ40p53(gUG2) constructs. Moreover, the construct P0-Δ40p53 was translated with a 3-fold lower efficiency compared with the P1-Δ40p53 transcript.

Subsequently, we compared ribosomal full-translation time values for three constructs with the same length of their 5′ non-coding regions, P1-Δ40p53, P1-Δ40p53(L) and P1-Δ40p53(S) (Table 1). The constructs differed substantially in their secondary structure arrangements surrounding the AUG1 codon (Fig. 4a). This codon is localized at the helix-bulge junction in P1-Δ40p53, in a large internal loop in P1-Δ40p53(L), whereas in P1-Δ40p53(S) it is embedded in the double-stranded structural context. The thermodynamic stability (ΔG) of hairpins G56-C169 present in these three constructs is −52.3 kcal/mol, −44.3 kcal/mol and −64.9 kcal/mol, respectively (Fig. 4a).

For P1-Δ40p53(S) the ribosomal full-translation time is longer compared to P1-Δ40p53 (8:00 min and 6:58 min), which means that the double-stranded structural environment of AUG1 is highly unfavourable for the ribosome binding and translation initiation. Translation efficiency of this construct is slightly decreased in comparison with P1-Δ40p53. The translation efficiency of P1-Δ40p53(L) is 1.7-fold higher than that of P1-Δ40p53. However, for unknown reasons, the full-translation time of the former construct is longer (7:35 min versus 6:58 min). Clearly, the structural environment of the AUG1 initiation codon has a large impact on ribosomal scanning and translation efficiency.

We prepared an additional construct, P1-Δ40p53(ΔA224), with nucleotide deletion in position 224, which resulted in the translation of the full-length luciferase only from codon AUG2 (we thank the Reviewer for this suggestion). The AUG1 codon is no longer in frame with luciferase and from that codon only a short peptide is synthesized. This product is not visible on an autoradiogram since it contains only a few radioactive methionine residues and probably it is quickly degraded in the reaction mixture (Suppl. Fig. S4). As only a single adenosine nucleotide was deleted from the single-stranded stretch we expect no changes in the folding of RNA P1-Δ40p53(ΔA224). Translation efficiency from AUG2 for this construct is comparable to the effectiveness of protein synthesis from AUG2 in RNA P1-Δ40p53 (Suppl. Fig. S4). The full-translation time value for P1-Δ40p53(ΔA224) is longer (7:27 min) than for the wild type P1-Δ40p53 (6:58 min), however, active luciferase is produced only from the second initiation codon.

#### mRNA constructs in which essential secondary structure elements of the 5′-terminal region of p53 mRNA are deleted

In order to establish how changes of selected structural elements of the 5′ non-coding region of p53 mRNA can influence translation initiation two mRNA constructs were assayed. In the first construct, P1-Δ40p53(Δ57), the characteristic hairpin motif G56-C169 was shortened. The hairpin has been shown to be present in several variants of native 5′-terminal region of p53 mRNA, also in variants with the coding region of different length, and in the full-length p53 mRNA^37,44^. In P1-Δ40p53(Δ57) the parental hairpin G56-C169 with ΔG of −52.3 kcal/mol was replaced by a smaller hairpin motif of much lower stability with ΔG of −18.4 kcal/mol. In the second construct, P1-Δ40p53(ΔHDM2), the relatively low stable U180-A218 hairpin with ΔG of −11.1 kcal/mol was deleted and replaced by a 3-nucleotide-long stretch. This hairpin has earlier been shown to be recognized by HDM2 protein^26^.

Translation efficiency of P1-Δ40p53(Δ57) construct is 2.4-fold higher than that of P1-Δ40p53 (Table 1). Unexpectedly, despite the fact that the transcript is shorter by 67 nucleotides, its full-translation time is substantially longer, amounting to 7:57 min versus 6:58 min. Also the deletion of hairpin U180-A218 in P1-Δ40p53(ΔHDM2) leads to a delay in the full-translation time by almost 2 minutes compared to the construct with wild-type 5′-terminal region of p53 mRNA. The translation efficiency is decreased to approx. 60% of that observed for P1-Δ40p53 (Table 1).

### Alterations in secondary structure elements of the 5′-terminal region of p53 mRNA impair the cap-independent translation from AUG2

**Figure 6:**
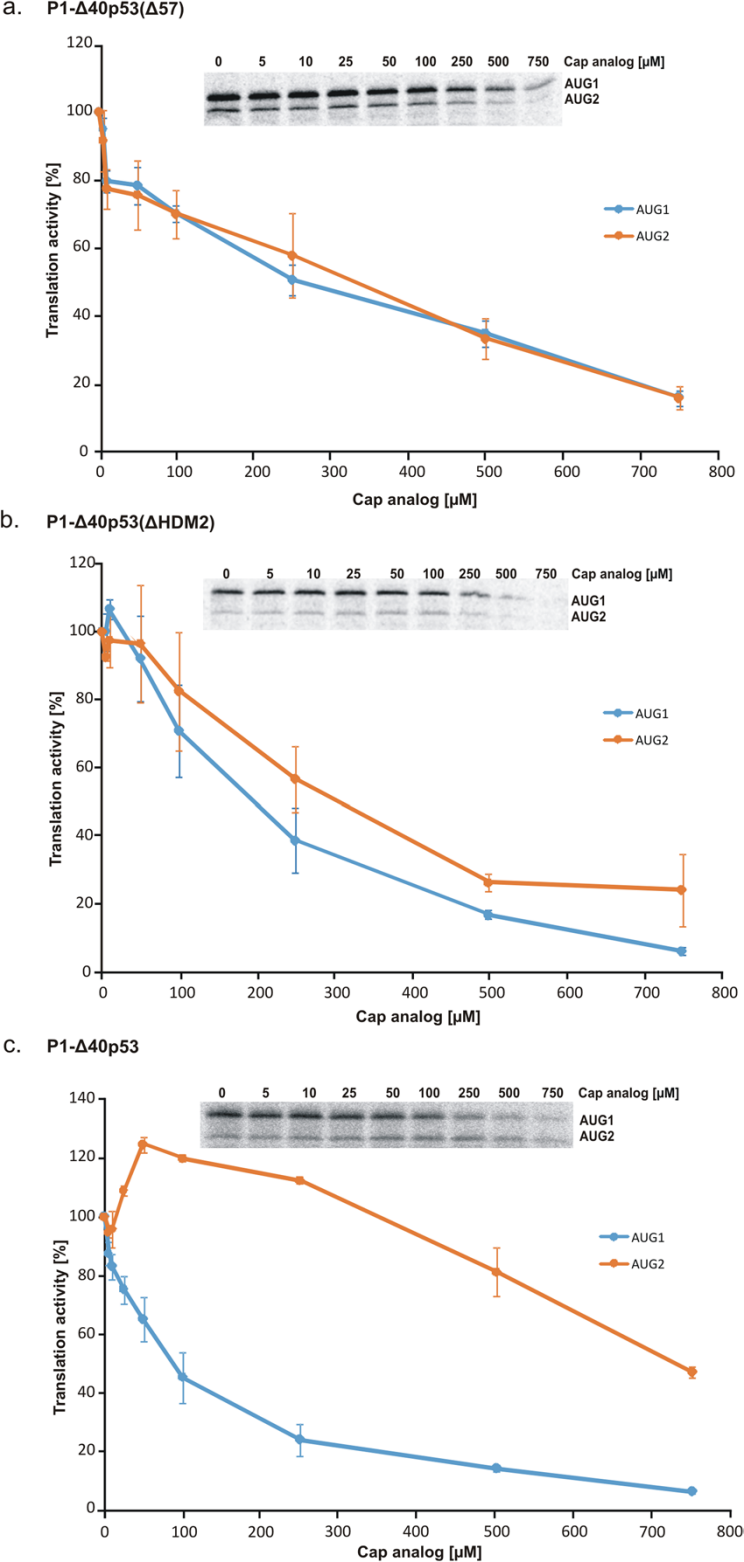
Translational inhibition of mRNA constructs with the cap analogue in RRL. The capped (**a**) P1-Δ40p53(Δ57), (**b**) P1-Δ40p53(ΔHDM2) and (**c**) P1-Δ40p53 mRNAs were translated in RRL in the presence of an increasing concentration of the cap analogue (m7GpppG) to inhibit cap-dependent translation. The amounts of protein products resulting from AUG1 and AUG2 initiation codons were determined, and following quantification and normalization to the values with no cap analogue added, they were displayed on the graph. The graph presents the mean of three independent measurements, with the standard deviations calculated and displayed on the diagrams. The full-length images of the gels were included in Suppl. Fig. S5.

Our previously published results have strongly suggested that translation of p53 protein from AUG1 is mainly cap-dependent, while the translation of Δ40p53 isoform from AUG2 shows a cap-independent character^37,44^. Here, we intended to elucidate the impact on translation of the two characteristic structural motifs present in the 5′-terminal region of p53 mRNA, hairpins G56-C169 and U180-A218. In order to determine whether the hairpins play a role in a cap-dependent process, we conducted translation of the P1-Δ40p53(Δ57) and P1-Δ40p53(ΔHDM2) constructs with an increasing concentration of the cap analogue. Both constructs were translated in RRL in the presence of ^35^S-methionine. The amounts of protein products, initiated from AUG1 and AUG2, were quantified and displayed as the function of the cap analogue concentration (Fig. 6a,b).

Surprisingly, the translation inhibition experiment conducted with the P1-Δ40p53(Δ57) construct with a shortened G56-C169 hairpin suggested a cap-dependent mechanism of translation initiation from both AUG1 and AUG2 codons. With the increase of the cap analogue concentration, the translation efficiency was gradually inhibited. Thus, it is of particular importance that after shortening of the G56-C169 hairpin the translation from AUG2 changed its characteristics from cap-independent to dependent on the presence of cap (compare Fig. 6a and Fig. 6c as well as Fig. 7 in Gorska *et al*.^37^).

**Figure 7:**
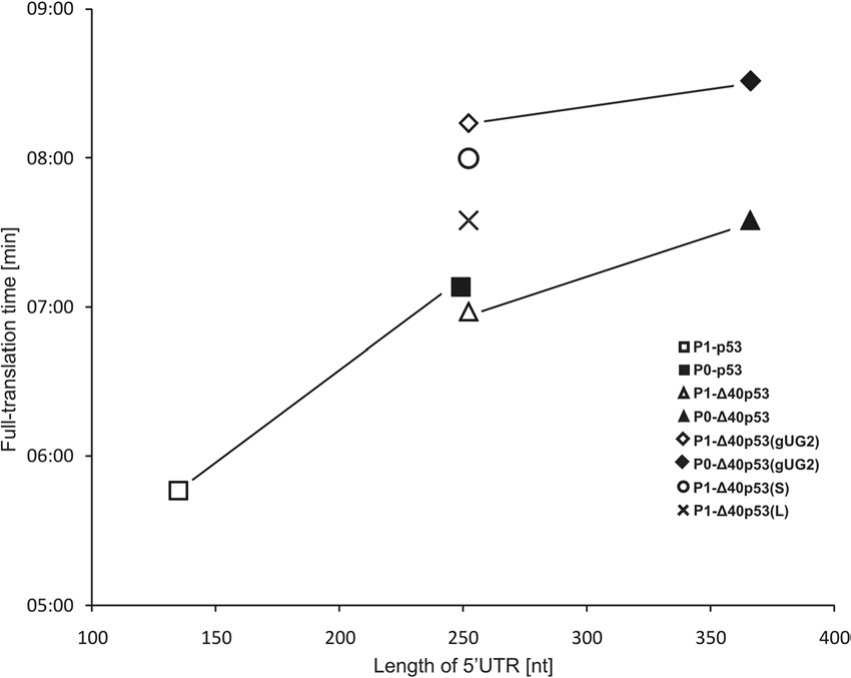
Dependence of the full-translation time for the model mRNA constructs on the length of their 5′UTRs. Symbols connected by lines represent the constructs which differ only in the region spanning P0 and P1 transcription promoters.

When the U180-A218 hairpin was removed in the P1-Δ40p53(ΔHDM2) construct, translation initiation from AUG2 also seemed to be cap-dependent (compare Fig. 6b and c as well as Fig. 7 in Gorska *et al*.^37^). At a low concentration of the cap analogue a slight increase of translation efficiency was, however, observed from both codons AUG1 and AUG2. The U180-A218 hairpin is important in p53 regulation because it has been shown as a binding site for HDM2 protein^26^.

The *in vitro* translation assay with an increasing concentration of the cap analogue was also performed for the P1-Δ40p53(ΔA224) construct, which produces the full-length luciferase only from codon AUG2 (Suppl. Fig. S4). The translation inhibition experiment suggested a cap-independent mechanism of translation initiation from AUG2 although the shape of the inhibition curve slightly differed from that observed for P1-Δ40p53 (Fig. 6c).

## Discussion

Over recent years, tremendous progress has been achieved in the elucidation of the translation details, its consecutive stages and factors involved in this process^1,45^. These studies have confirmed that translation initiation is a stage at which translation efficiency of various mRNAs is differentiated and at which most events leading to translation regulation occur. Recently, the composition and structure of the initiation complex has been elucidated^46^. Despite recent achievements in the field, the step between recognition of the cap structure and the formation of the full ribosome, from the 1980s known as the scanning process, is still not well understood. Importantly, it has been believed that while the scanning complex moves along the 5′ non-coding region, the structural and functional features of this region i.e. stable secondary structure motifs or IRES elements may contribute to translation. Moreover, beside standard translation factors, several proteins interacting with the 5′ non-coding region of mRNA, seem to take part in translation regulation^1,8,31,33^.

In this work we focused on the process of ribosomal scanning of several variants of the 5′ non-coding region of p53 mRNA. The variants were a consequence of transcription initiation from different promotor sites P0 and P1 as well as translation initiation from two initiation codons AUG1 and AUG2. Moreover, in two model mRNA constructs we changed the structural context of AUG1 codon placing the codon in a large internal loop or in a double-stranded stem. Finally, in two other constructs, hairpin motifs characteristic to this region were changed. The G56-C169 hairpin was shortened or the U180-A218 hairpin was deleted. The variants of the 5′ non-coding region of p53 mRNA were attached to the sequence encoding the reporter luciferase and the translation activity of such model mRNA constructs was assayed in rabbit reticulocyte lysate system. It is believed that cell lysates are deprived of most regulatory proteins^47^, therefore such conditions are well suited to evaluate the effect of RNA structure on translation. Importantly, the secondary structures of the 5′ terminal regions of the studied mRNA constructs were mapped experimentally in solution by biochemical methods (Suppl. Fig. 2a–e and our earlier data^37,44^). This allowed correlation of the secondary structure of the 5′ non-coding region of p53 mRNA with this region functioning in translation. Structural differences were correlated with various scanning rates and efficiencies of reporter protein synthesis.

The diagram shown in Fig. 7 illustrates how changes in the 5′ non-coding region of p53 mRNA influence ribosomal scanning of that region. In particular, for two pairs of constructs bearing only one initiation codon AUG1, P0-p53 – P1-p53 and P0-Δ40p53(gUG2) – P1-Δ40p53(gUG2), the values of full-translation time are higher for the P0-initiated constructs. These constructs have additional 113-nucleotide-long sequence stretches at their 5′ ends. The difference in the full-translation time is, however, different for both pairs of constructs and corresponds to the scanning rates of these 113-nt stretches of 1.4 nt/s and 6.6 nt/s, respectively. These regions are folded into very different secondary structures which seems to be responsible for the different scanning rates. Our observation contradicts earlier suggestions that the secondary structure of the scanned region does not influence its scanning rate^18^.

For two constructs P0-Δ40p53 and P1-Δ40p53 with two initiation codons AUG1 and AUG2 the values of full-translation times are longer than for the same constructs bearing only one initiation codon AUG1 (Fig. 7). Likely, this is an effect of synthesis of an additional protein initiated from the AUG2 codon. In the luminescence assay, this protein contributes to the observed values of full-translation time. When comparing P0-Δ40p53 and P1-Δ40p53 constructs, the scanning rate of an additional 113-nt stretch of the longer transcript of approx. 3.0 nt/s was calculated. It was in the same range as the values of 1.4 nt/s and 6.6 nt/s, determined for the other two pairs of constructs. The scanning rates calculated in our study are comparable to earlier values of ∼6 and 8 nt/s determined for the Krebs-2 (30 °C) and wheat germ (25 °C) cell-free translation systems^18^ or to the estimate of ∼10 nt/s in yeast translation extract^48^.

It has to be noted that translation efficiencies of the P0-p53 and P1-p53 constructs differ considerably and the former construct is translated with a several-fold lower efficiency (Fig. 3b and Table 1). The double-stranded secondary structure of the construct in the 5′-terminal region is likely to be responsible for this effect. The presence of stable structural elements downstream in the ORF regions of mRNAs seems to have a smaller impact on translation. Similar observations have earlier been reported by other authors^49^.

A strong impact of the structure of the hairpin motif in which the initiation AUG codon is embedded on the translation efficiency has been observed in *Procaryota*^9,10^. An increase of the hairpin stability (ΔG) by about −1.4 kcal/mol caused the reduction of the initiation translation rate of bacteriophage coat protein by a factor of 10. Moreover, it was shown that it is the secondary structure rather than sequence composition that determines translation efficiency. Importantly, based on free energy calculations, de Smit and van Duin suggested that translation efficacy was related to the fraction of an unfolded RNA region of the ribosome binding site^10^. These suggestions are in line with our observations of different translation productivity for P1-Δ40p53, P1-Δ40p53(S) and P1-Δ40p53(L) (Table 1 and Fig. 2). Differences in the efficiency of protein synthesis nicely correspond to the ΔG values of the G56-C169 hairpins in these constructs: −52.3 kcal/mol, −64.9 kcal/mol and −44.3 kcal/mol, respectively (Fig. 4a). It has also been demonstrated in yeast model that eIF4G promotes mRNA unwinding in order to select AUG codon and re-initiation^50^. Therefore, structural RNA elements within the ribosome binding site need to be unfolded for translation initiation^9,10,50^. This might explain the differences in the translation efficiency of RNA constructs with various 5′-terminal regions of p53 mRNA, in which the initiation codons are located in different structural environments (Table 1).

Thermodynamic stabilities of most hairpin motifs found in our constructs’ 5′ non-coding regions are no stronger than approx. −20 kcal/mol. The only hairpin motif of higher stability is that of G56-C169 with ΔG of approx. −50 kcal/mol. However, the hairpin does not contain a perfect double-stranded stem but it is divided into a few segments interspaced by internal loops or bulges. Undoubtedly, unfolding such structure is much easier than unfolding a perfectly base-paired stem. Thus, this imperfect hairpin impacts translation to a lower extent than it could be expected based on its thermodynamic stability only. Some earlier data have suggested that hairpins with ΔG lower than −50 kcal/mol may still be efficiently translated as long as the stem GC content is relatively low^49^.

It has been shown that a hairpin with ΔG of −30 kcal/mol located 5 nt from the 5′ end of a model mRNA significantly lowers its translation efficiency in RRL while a hairpin with ΔG of −13 kcal/mol almost does not influence translation^51^. Hairpin motifs located at a larger distance from the 5′ end influence translation to a smaller degree. In general, inhibition has been observed for hairpins with ΔG below −30 kcal/mol. This energy value may only be used as a suggestion since the final effect also depends on other structural or cellular factors. For example, insertion of a GC-rich stem-loop motif that directly involves the initiation AUG codon with ΔG −30 kcal/mol in the middle of 5′UTR has not inhibited the initiation of translation in COS cells^11^. Similarly, the same hairpin introduced to another transcript has not substantially impacted the scanning rate of LINE-1 5′UTR^18^. On the other hand, strong inhibition of translation in RRL has been observed for a transcript with a hairpin with ΔG of −30 kcal/mol when the initiation codon was embedded in a double-stranded region of the hairpin^51^. Generally, elements of the RNA secondary structure present in 5′ non-coding regions show a stronger impact on translation when they are located close to the 5′ terminus of mRNA or the AUG initiation codon is embedded in their structures. This conclusion is consistent with our observation regarding the translation of variants of p53 mRNAs in RRL.

Structural environment of initiation codon AUG1 in the model mRNA constructs has a large influence on the full-translation time and translation efficiency. Comparison of full-translation time values for P1-Δ40p53(S) and P1-Δ40p53(L) with that determined for P1-Δ40p53 shows that both mutated sequences are scanned at a slower rate (Fig. 4a and Table 1). The longest full-translation time value was calculated for P1-Δ40p53(S) in which the initiation codon is embedded in a double-stranded stem. This shows that hairpin G56-C169 constitutes a remarkable barrier for the scanning complex. In P1-Δ40p53(L) unfolding of the mutated hairpin with a large internal loop is undoubtedly easier than with the double-stranded stem in which the AUG1 codon is placed. Moreover, the translation efficiency of this construct is the highest which may be explained by its shorter full-translation time and a smaller probability of the fall-off effect for the complex and/or better accessibility of AUG1 to translation machinery. The above results show again that the ribosomal scanning time is not always directly proportional to the length of 5′UTR and that the structural context in which the initiation codon is located is crucial for translation initiation.

A strong impact of the AUG1 structural environment is also seen when the translation of P1-Δ40p53(Δ57) and P1-Δ40p53(ΔHDM2) is compared. Despite the almost identical length of their 5′UTRs, P1-Δ40p53(Δ57) is translated with a several-fold higher efficiency, similar to that observed for P1-p53 (Fig. 5a and Table 1). However, in both constructs the AUG1 codon is not embedded in the large G56-C169 hairpin, which is a characteristic element of native p53 mRNA. Thus, this hairpin greatly contributes to the low translation efficiency of this mRNA, unlike hairpin HDM2, the removal of which does not result in an increase of translation efficiency. It might be even expected that the presence of hairpin HDM2 located downstream of AUG1 would strengthen translation. Such effect has been observed for stable hairpin motifs located downstream of initiation codons. They are supposed to slow the movement of the scanning complex, increase the probability of AUG codon recognition and initiation of polypeptide synthesis^2,52^.

Furthermore, it has been shown that the region spanning the two initiation codons AUG1 and AUG2 is sufficient for cap-independent translation initiation^36^. Interestingly, an increase in the translation efficiency of P1-Δ40p53(Δ57) from AUG2 (Fig. 2) supports the hypothesis that the G56-C169 hairpin is mostly responsible for blocking cap-dependent translation to promote IRES activity and protein synthesis from the second initiation codon. On the other hand, a decrease in the translation efficiency of P1-Δ40p53(ΔHDM2) from AUG2 is in line with the suggestion that U180-A218 hairpin is the structural element where IRES activity would be placed. It is important that for mRNAs with IRES elements two types of the translation initiation mechanism may operate simultaneously. Both mechanisms, with and without scanning the 5′ untranslated region, may to some extent influence the full-translation time values which were determined experimentally in this study.

Additionally, the P1-Δ40p53(Δ57) and P1-Δ40p53(ΔHDM2) constructs were also tested in an *in vitro* translation assay in the presence of an increasing concentration of the cap analogue. Our earlier data for the mRNA constructs with native p53 5′UTR has suggested that translation from AUG1 codon is a cap-dependent process while translation from AUG2 is cap-independent and is probably IRES driven^37,44^. For the P1-Δ40p53(Δ57) and P1-Δ40p53(ΔHDM2) constructs experimental curves are similar for translation initiated from both codons AUG1 and AUG2 suggesting that translation occurs in a cap-dependent mode (Fig. 6). This result exemplifies the importance of both structural elements, hairpins G56-C169 and U180-A218, for the functioning of the 5′-terminal region of p53 mRNA as an IRES element. It turned out that manipulation of the secondary structure of this region changed the mechanism of translation initiation.

In this work we describe how the length and structural differences in variants of 5′UTR of p53 mRNA influence the two crucial parameters characterizing translation, namely the scanning rate and translation efficiency. Variants of p53 5′UTR clearly exemplified the complex character of the impact of this region’s features on translation. Structural factors connected with the length of 5′UTR, the presence of stable secondary structure motifs, the structural environment of initiation codons and IRES elements have a decisive impact on translation efficiency. Moreover, structural elements of this region have been shown to be platforms for binding of several proteins *in vivo*, forming a complex RNA-protein regulatory system. We believe that our data will be helpful in further studies aimed at unveiling the mechanisms of regulation of p53 synthesis.

## Materials and Methods

### Synthesis of DNA templates

The dsDNA templates for the constructs: P1-p53, P0-p53, P1-Δ40p53 and P0-Δ40p53 were obtained previously in our laboratory^33^. The dsDNA template for construct P1-Δ40p53(ΔHDM2) was obtained by cleaving pRL-CMV plasmid vector carrying P1-Δ40p53 insert with Xba I and Csp 45I restriction enzymes (Thermo Scientific), according to the manufacturers’ protocol. Then the ΔHDM2 insert 5′-AAGTCTAGAGCCACCGTCCAGGG AGCAGGTAGCTGCTGGGCTCCGGGGACACTTTGCGTTCGGGCTGGGAGCGTGCTTTCCACGACGGTGACACGCTTCCCTGGATTGGCAGCCAGACTGCCTTCCGGGTCACTGCCATGGAGGAGCCGCAGTCAGATCCTAGCGTCGAGCCCCCTCTGAGTGAAAACAACGTTCTGTCCCCCTTGCCGTCCCAAGCAATGGATTCGAATAG-3′ was ligated into the vector using T4 DNA Ligase (Thermo Scientific), according to the manufacturers’ protocol. Afterwards, the dsDNA construct was transformed into *E. coli* DH5α competent cells (Invitrogen). The sequence of the dsDNA construct was confirmed by sequencing.

The dsDNA template for construct P1-Δ40p53(Δ57) was obtained as follows: two DNA oligomers F(Δ57)1: 5′-GGCTAGAGCCACCGUCCAGGGAGCAGGTAGCTGCT GGGCTCCGGGGACACTTTGCGCTTTCCACGAC-3′, and R(Δ57)1: 5′-CTCAGAGGGG CTCCTCCATGGCAGTGACCGGGAAGCGTGTCACCGTCGTGGAAAG-3′ were used for synthesis of the molecule. Equimolar amounts of both oligomers were annealed and a double-stranded DNA template was generated by PCR. The PCR reaction contained: 200 µM of forward and reverse primers, 75 mM Tris-HCl pH 8.8, 20 mM (NH_4_)_2_SO_4_, 0.01% Tween 20, 0.2 mM of each dNTP, 1.5 mM MgCl_2_ and 30 U/ml *Taq* polymerase (Fermentas). The reaction was conducted for 8 cycles of initially 2 min of denaturation at 95 °C, then 30 s at 95 °C, 30 s at 72 °C and 1 min at 72 °C, and finally 5 min at 72 °C. The reaction product was purified using PCR purification columns (Eurx), and the obtained DNA was used as a template for the next PCR reaction. Primers: F(Δ57)2: 5′-CTTTCCACGACGGTGACACGCTTCCCGGTCACTGCCATGGAGGAGCCCCTCTGAG-3′ and R(Δ57)2: 5′-CATTGCTTGGGACGGCAAGGGGGACAGAACGTTGTTTTCAGGAAGTAGTTTCCATAGGTCTGAAAATGTTTCCTGACTCAGAGGGGGC-3′ were used and PCR conditions were as described above, with 3 nM template and 0.25 mM concentration of the primers. After purification the dsDNA was ligated to the pRL-CMV plasmid vector (Promega) using T4 DNA ligase (Thermo Scientific), according to the manufacturers’ protocols. Afterwards, the dsDNA constructs was transformed into *E. coli* DH5α competent cells (Invitrogen). The sequence of the dsDNA construct was confirmed by sequencing.

### Site directed mutagenesis

The dsDNA templates for the constructs: P1-Δ40p53(L), P1-Δ40p53(S) and P1-Δ40p53(gUG2) were obtained by site-directed mutagenesis of the P1-Δ40p53 dsDNA construct, whereas for the P0-Δ40p53(gUG2) construct P0-Δ40p53 was used as a template. The following forward (F) and reverse (R) primers were used: F(L): 5′-GGCTGGGAGCGTGCTTAAGCCGACGGTGACACGCTT-3′, R(L): 5′-AAGCGTGTCACCGTCGGCTTAAGCACGCTCCCAGCC-3′, F(S): 5′-CGTGCTTTCCATGGCGGTGACACGCTTC-3′, R(S): 5′-AAGCGTGTCACCGCCATGGAAAGCACG-3′, F(gUG2): 5′-TGCCGTCCCAAGCAGTGGATTCGAAAGTT-3′ and R(gUG2): 5′-AAACTTTCGAATCCACTGCTTGGGAC GGCAA-3′. The PCR reaction contained: 0.5 ng of the dsDNA template, 75 µM forward or reverse primer, 0.2 mM of each dNTP, 20 mM Tris-HCl pH 8.8, 10 mM KCl,10 mM (NH_4_)_2_SO_4_, 2 mM MgSO_4_, 0.1% Triton X-100, 0.1 mg/ml BSA and 1.5 U of *Pfu* polymerase (Promega) in the final volume of 50 µl. Reactions with forward primer and reverse primer were performed separately, but in the same conditions for 10 cycles of initially 2 min of denaturation at 95 °C, then 50 s at 95 °C, 50 s at 60 °C, 16 min at 68 °C, and finally 7 min at 68 °C. Afterwards, two reactions were combined, 1.5 U of *Pfu* polymerase was added, and PCR reaction was performed in the same conditions as above, for 20 cycles. Subsequently, the reaction products were purified by phenol/chloroform extraction (1:1) and precipitated with ethanol. Then, 5 µg of each dsDNA sample was treated with 50 U of Dpn I restriction enzyme (Thermo Scientific), according to the manufacturer’s protocol. After digestion, dsDNA was transformed into *E. coli* DH5α competent cells (Invitrogen) and the sequence of the construct was confirmed by sequencing.

### *In vitro* transcription and translation

All dsDNA templates were first linearized with Not I or Xba I restriction enzymes (Thermo Scientific), in accordance with the manufacturers’ protocol. Subsequently, the *in vitro* transcription was performed using AmpliScribe T7 *In Vitro* Transcription Kit (Epicentre). All RNA constructs were synthesized in the reactions containing 3 mM Anti Reverse Cap Analog (ARCA, Epicenter Biotechnologies), 1.5 mM GTP, and 7.5 mM of the remaining nucleotide substrates. After the transcription reactions the RNA probes were treated with DNase I enzyme for 20 min at 37 °C and purified using RNA Clean-up and Concentration Kit (Norgen).

*In vitro* translation was performed in Rabbit Reticulocyte Lysate (RRL) System (Promega)^33^. The reaction contained 8.75 µl RRL, 20 µM aminoacid mixture minus methionine, 0.5 µl ^35^S-methionine (1000 Ci/mol, Hartman Analytic), 10 U of RiboLockRNase inhibitor (Thermo Scientific) and 1.25 pmol of capped RNA which was previously denatured for 5 min at 65 °C. The final volume of the reaction was 12.5 µl and it was conducted for 90 min at 30 °C. Afterwards, the reaction was treated with 0.16 µg of RNase A for 5 min at 20 °C, and denatured for 2 min at 80 °C in the presence of SDS Sample Buffer and 100 mM DTT. The reaction products were analysed in 15% SDS-PAGE, followed by radioisotope imaging with FLA 5100 image analyser (Fuji).

As far as the translation inhibition assay is concerned, RRL was pre-incubated with an increasing concentration of m7GpppG cap analogue from 0 to 750 μM (Epicenter Biotechnologies) and the same concentrations of magnesium acetate, for 15 min at 30 °C. Afterwards, 20 µM aminoacid mixture minus methionine, 0.5 µl ^35^S-methionine (1000 Ci/mol, Hartman Analytic), 10 U of RiboLockRNase inhibitor (Thermo Scientific) and 1.25 pmol of previously denatured RNA were added to the RRL samples and incubated for 90 min at 30 °C. The samples were treated with RNase A and analysed in 15% SDS-PAGE as in the regular translation protocol described above.

### *Renilla* Luciferase Reporter Gene assay

Real-time translation was performed in 17.5 µl of RRL (Promega), 20 µM of aminoacid mixture minus methionine, 20 µM of aminoacid mixture minus leucine, 10 U of RiboLockRNase inhibitor (Thermo Scientific), 1.25 pmol of capped RNA, previously denatured for 5 min at 65 °C and 25 µM of coelenterazine (Promega) in the final volume of 25 µl. The samples were placed in the Victor 4 Spectrophotometer (Perkin Elmer) which maintained the temperature at 30 °C. Luminescence intensity was measured every 8 s for 1 s, and 140 such measurements were performed. Calculations of full-translation time were performed using the *Mathematica* 8.0 software (Wolfram Research, Inc., Champaign, Il, USA) by the second derivative method in a similar way as described in Vassilenko *et al*.^18^. In order to reduce the effect of noise the original kinetic profiles of luminescence signal were first approximated by a polynomial function and then differentiated to obtain the maximum corresponding to the full translation time (for detailed description see Supplementary Information). Full-translation time with standard deviation was calculated for each construct based on three independent experiments. Moreover, the maximal luminescence activity values with standard deviations were calculated for each construct and the values were normalized to the value obtained for the P1-Δ40p53 variant.

## Electronic supplementary material

Supplementary Information
