# Supplementary material for: Variants of the 5′-terminal region of p53 mRNA influence the ribosomal scanning and translation efficiency
Source: Sci Rep. 2018 Jan 24;8:1533. doi: 10.1038/s41598-018-20010-2 (PMC5784139; doi:10.1038/s41598-018-20010-2)
Supplement: Supplementary file 1 — Supplementary Information [file 41598_2018_20010_MOESM1_ESM.pdf]

Supplementary Information

Variants of the 5′-terminal region of p53 mRNA influence the ribosomal scanning and translation efficiency

Paulina Zydowicz-Machtel[1§], Agata Swiatkowska[1§], Łukasz Popenda[2], Agnieszka Gorska[1]& Jerzy Ciesiołka[1*]

[1]Institute of Bioorganic Chemistry, Polish Academy of Sciences, Noskowskiego 12/14, 61-704 Poznan, Poland
[2]NanoBioMedical Centre, Adam Mickiewicz University in Poznan, Umultowska 85, 61-614 Poznan, Poland

[§]Paulina Zydowicz-Machtel and Agata Swiatkowska contributed equally to this work

*Correspondence and requests for materials should be addressed to Jerzy Ciesiołka (email: jerzy.ciesiolka@ibch.poznan.pl)

Supplementary Methods

Pb^{2+}-induced RNA cleavage

Prior to the Pb^{2+}-induced RNA cleavage70 pmol of RNA was denatured in the buffer containing 40 mM NaCl, 10 mM Tris-HCl pH 7.2, 10 mM $MgCl_2$ for 5 min at 65°C and then renatured for 5 min at 37°C. Afterwards, the reaction was divided into four samples and lead acetate solution was added to each sample to the final concentration of 0.5 mM, 1 mM, 2 mM, and an equal volume of water was added to the control reaction. The final volume of the reaction was 10 µl and it was conducted for 3 min at 37°C. After termination by an addition of 10 mM EDTA, the RNA was precipitated with 300 mM sodium acetate pH 5.2, 0.2 mg glycogen (Thermo Scientific) and 180 µl of ethanol. The RNA pellet was dissolved in 10 µl of H_2O and used in primer extension reaction.

For reverse transcription reaction 2 µl of RNA sample and 2 pmol of [^{32}P]-labelled DNA Rdlug primer: 5′-TCCATTGCTTGGGACGGCAAGG-3′ were first denatured for 5 min at 65°C, in the final volume of 10 µl H_2O. The reaction mixture contained: 50 mM Tris-HCl pH 8.3, 75 mM KCl, 3 mM $MgCl_2$, 6 mM DTT, 200 U SuperScript III Reverse Transcriptase (Invitrogen), 0.5 mM dNTPs Mix, and 10 U RiboLockRNase inhibitor (Thermo Scientific). The final volume of the reaction was 20 µl and it was conducted for 1 h at 55°C followed by 15 min at 70°C. Simultaneously, sequencing of the RNA was performed, in order to assign the cleavage sites. The sequencing reaction was prepared in the same conditions as described above, using RNA samples and 0.2 mM dideoxy-terminating nucleotides. Subsequently, 1 µl

of 4 M NaOH was added to each sample, incubated for 5 min at 95°C, then the samples were put on ice and 160 mM Trizma was added. Finally, the solution containing 8 M urea, 20 mM EDTA and xylene cyanol dye was added to the samples, incubated for 3 min at 95°C and loaded on 8 M urea, 8% polyacrylamide gels. After electrophoresis the gels were scanned by phosphorimaging using the FLA 5100 image analyser (Fuji).

SHAPE analysis

The reaction mixture containing 20 pmol of RNA (0.4 µM final concentration) and the renaturation buffer: 10 mM Tris pH 8.0, 100 mM KCl, and 0.1 mM EDTA, was heated at 90°C for 3 min and slowly cooled (0.1°C/s) to 4°C. The folding buffer: 40 mM Tris pH 8.0, 5 mM $MgCl_2$, 130 mM KCl, and 0.1 mM EDTA was added followed by water to a final volume of 146 µl. The sample was incubated at 37°C for 10 min and separated into two reactions. The RNA solution was mixed with 7.3 µl of 55 mM NMIA (Invitrogen) in DMSO (5.5 mM final concentration). The control reaction contained DMSO without NMIA. Both reactions were incubated for 50 min at 37°C and the RNA was precipitated with 300 mM sodium acetate pH 5.2, 1 µl glycogen (20 mg/ml), and 3 volumes of ethanol. After centrifugation, the RNA was resuspended in water. Analysis of the modification sites was performed by the primer extension reaction, in the same manner as for Pb^{2+}-induced RNA cleavages.

Determination of full-translation time

It is worthy of note that the expression 'full-translation time' actually refers to 'full-translation time of first translation rounds'. Once several ribosomes are placed on the mRNA the original structures will rather not be formed again, so the first round of translation usually takes longer because the RNA is still structured at that time. However, the term 'full-translation time' is used in the manuscript for simplification.

Determination of full translation time was carried out using the second derivative maximum method in *Mathematica* 8.0 software (Wolfram Research, Inc., Champaign, Il, USA). Example data on luminescence activity obtained from the *Renilla* luciferase assay *in vitro*, in rabbit reticulocyte lysate (RRL) as a function of time collected for P0-Δ40p53 construct are shown in Figure S1a.

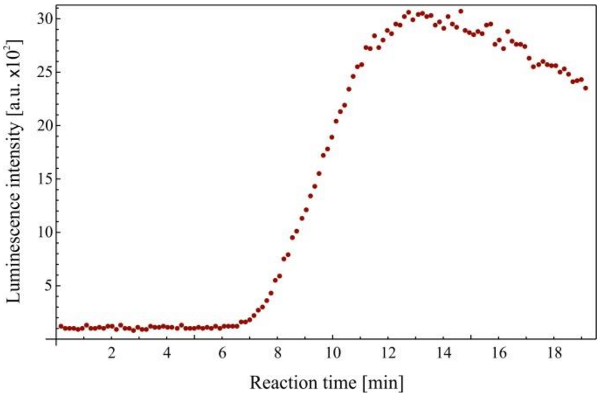

Figure S1a. Data on luminescence activity obtained from the *Renilla* luciferase assay *in vitro*, in RRL as a function of time. The height of the peak reflects translation efficiency from the P0-Δ40p53

construct. A kink at the initial rise of the curve corresponds to the moment of appearance of luciferase activity and represents the completion of protein synthesis (i.e. combined duration of the initiation, elongation and termination) indicating the average translation time.

In general, in the case of data obtained directly from the experiment, substantial noise or inaccuracy (i.e. experimental artefacts caused by the interfering phenomena, instrumental distortion, faulty calibration, etc.) could be observed. Conventional techniques for numerically calculating derivatives greatly amplify any noise or distortion present in the data what often makes the results illegible. The application of noise reduction techniques on data before or after differentiating can lead to unsatisfactory results. Therefore with regard to noisy or error-contaminated data, their prior approximation by an analytic function appears to be far more efficient. For the example given in Figure S1a,the signal-to-noise ratio is acceptable. However, the intensity of luminescence signal is not so high in all recorded data. Consequently, the level of noise in those cases is much higher.

It is known that certain relationships of common physical and chemical processes represented by the experimental data sets can be expressed or approximated by a mathematical function. To represent the data we can use a specific formula if a theoretical model of a given phenomenon is present or we can try to use a different form of function (e.g. polynomial, trigonometric, logarithmic or exponential) to empirically obtain the most efficient approximation. Among a number of tested models, a polynomial function turned out to be appropriate to describe the experimental data. Polynomials belong to the simplest yet very important class of analytical functions which is a suitable choice in many cases. Therefore, during the first step of analysis, the original kinetic profiles of the luminescence signal were fitted by a polynomial function (Figure S1b) which in general may be represented by:

$$f(x) = a_0x^0 + a_1x^1 + a_2x^2 + \cdots + a_nx^n = \sum_{i=0}^{n} a_ix^i \quad (1)$$

where a_0, a_1, a_2, up to a_n are the fitting parameters and n is the order of the polynomial. In our studies $f(x) = L(t)$ when the order of the polynomial n is equal to 17. The degree of the polynomial function has been selected so as to get the best convergence to the experimental data. The R^2 coefficient was the decisive factor during the evaluation process.

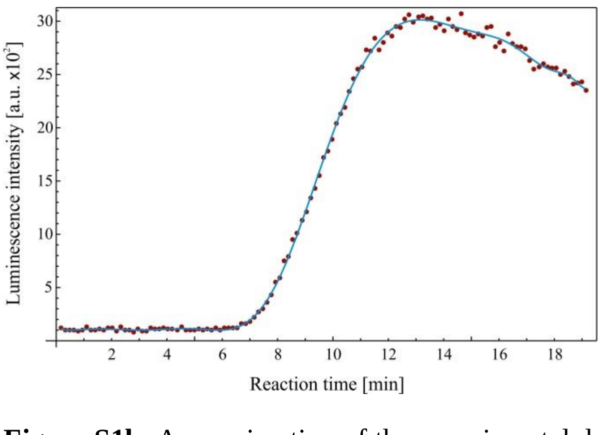

Figure S1b. Approximation of the experimental data (red dots) by a polynomial function (blue line).

In the next two steps, the polynomial function fitted to the luminescence curve was differentiated. The first derivative is presented in Figure S1c and the second one – in Figure S1d.

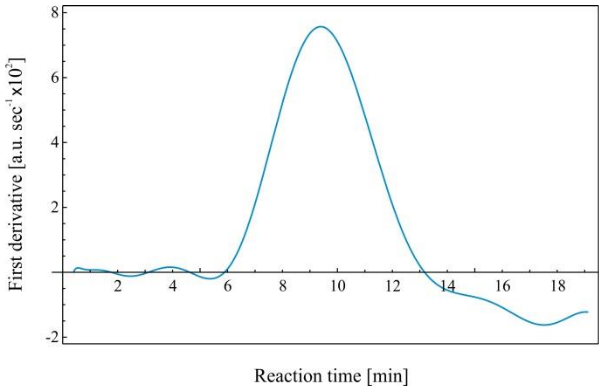

Figure S1c. First derivative of the polynomial function shown in Figure S1b.

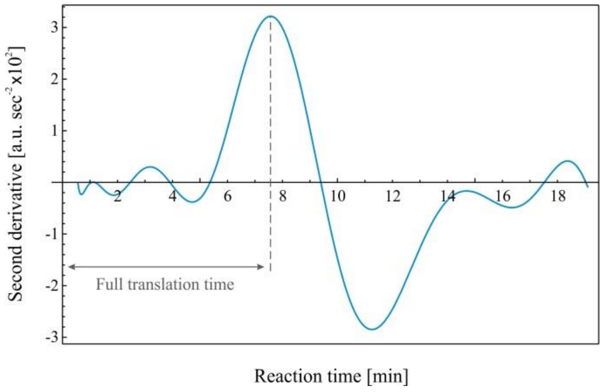

Figure S1d. Second derivative of the function fitted to luminescence signal measured for the P0-Δ40p53 construct. The marked position (position of the maximum of the derivative) corresponds to the average full-translation time.

The argument for which the second derivative assumes the maximum value determines the average full-translation time or the average time for synthesis of full-length luciferase in a given translation round. In the presented example (Figures S1a-d), the average full-translation time for the translation of the P0-Δ40p53 construct was 7 min 36 s. The goodness of fit, R^2 was 0.999664. To verify the reproducibility of data (obtained as above), three independent data sets were collected for each construct. Upon these measurements, standard deviations of the data were also determined.

Supplementary Figure S2: Secondary structure probing of the 5′-terminal regions of the studied mRNA constructs. Five mRNA variants with various 5′-terminal regions of p53 mRNA located upstream of the *Renilla* luciferase coding sequence were prepared. The RNA secondary structure models of these regions were predicted by *RNAstructure* software based on the results obtained from Pb^{2+}-induced cleavage and SHAPE mapping methods.

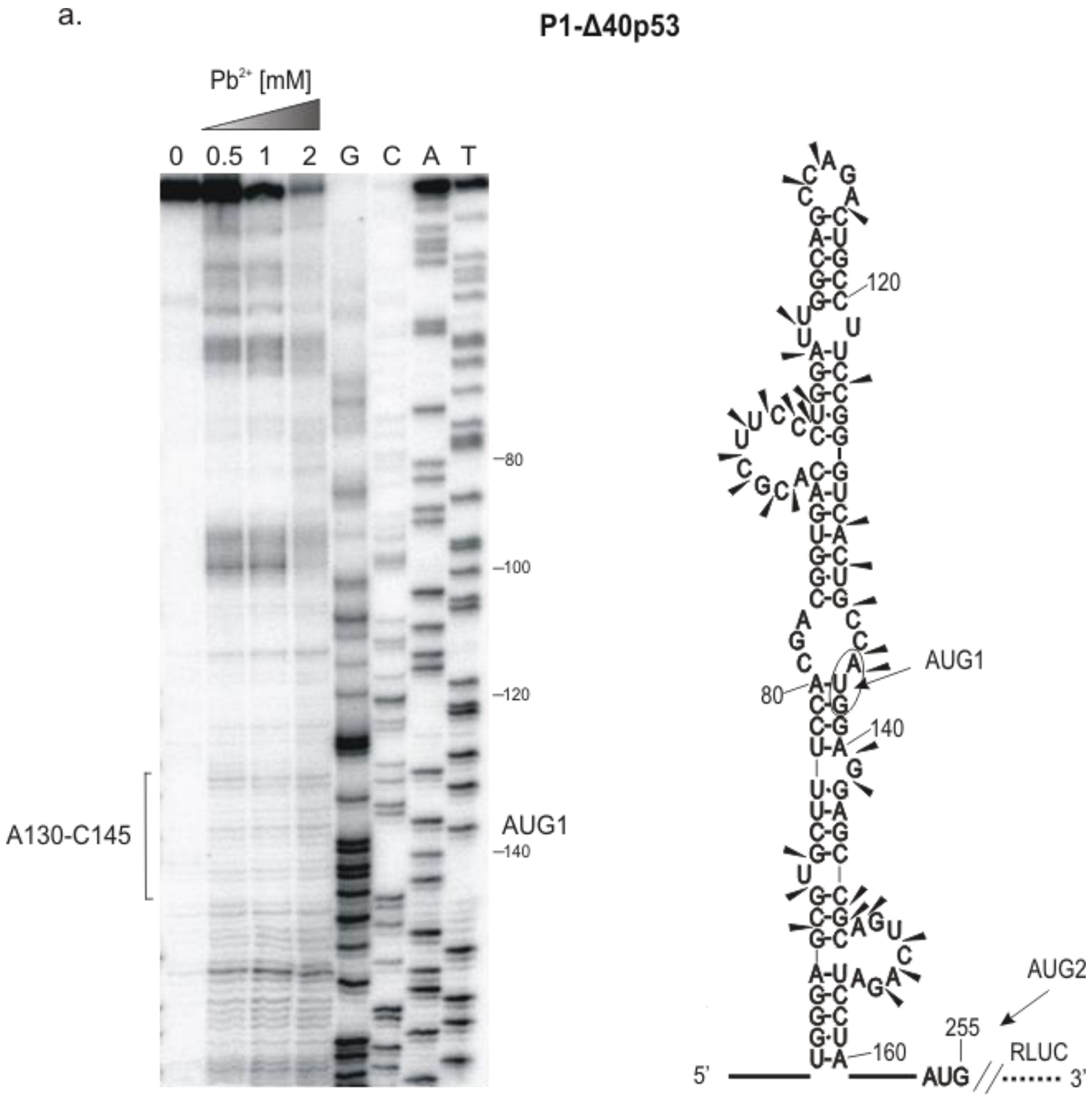

(a) Analysis of the structure of the mRNA construct P1-Δ40p53 using the Pb^{2+}-induced cleavage method. On the left, the results of the Pb^{2+}-induced cleavage reaction are presented. Lanes G, C, A and T indicate the reaction with guanosine, cytosine, adenine and thymine dideoxy nucleotides respectively, whereas 0, 0.5, 1, and 2 mM indicate the increasing concentration of lead(II) acetate solution. Selected nucleotide positions are marked on the right side of the autoradiogram. The region with AUG1 codon is marked on the gel as A130-C145. On the right, a simplified secondary structure model of mRNA construct is presented. Initiation codon AUG1 is circled, whereas Pb^{2+}-induced cleavages are denoted by black arrows.

b.

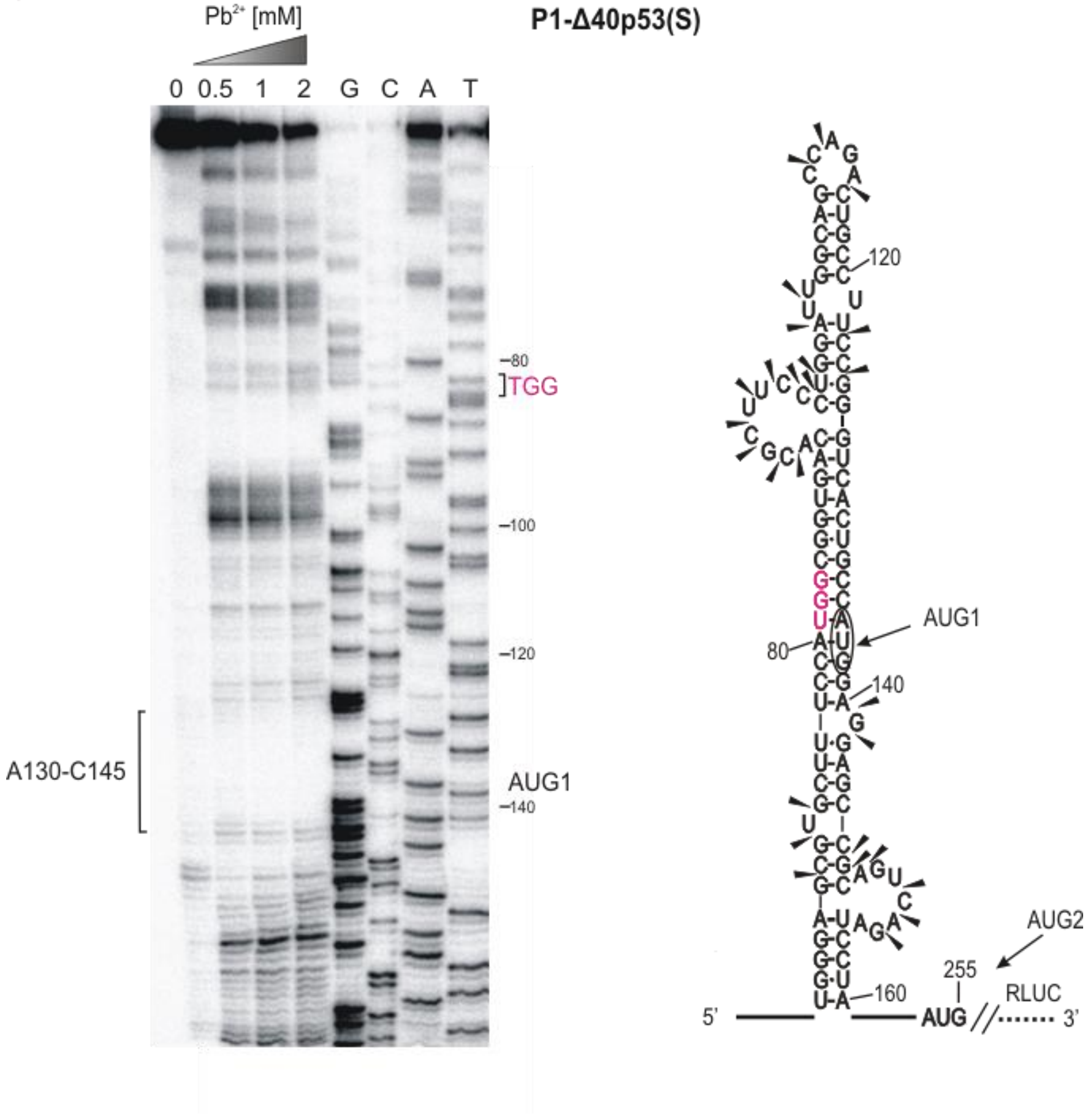

(b) Mapping of secondary structure of the P1-Δ40p53(S) construct by Pb^{2+}-induced cleavage approach. The AUG1 codon is located in double-stranded region. Mutated nucleotides are marked in red on the gel and on the secondary structure model. Description of the gel and structural model is analogous to that in panel a.

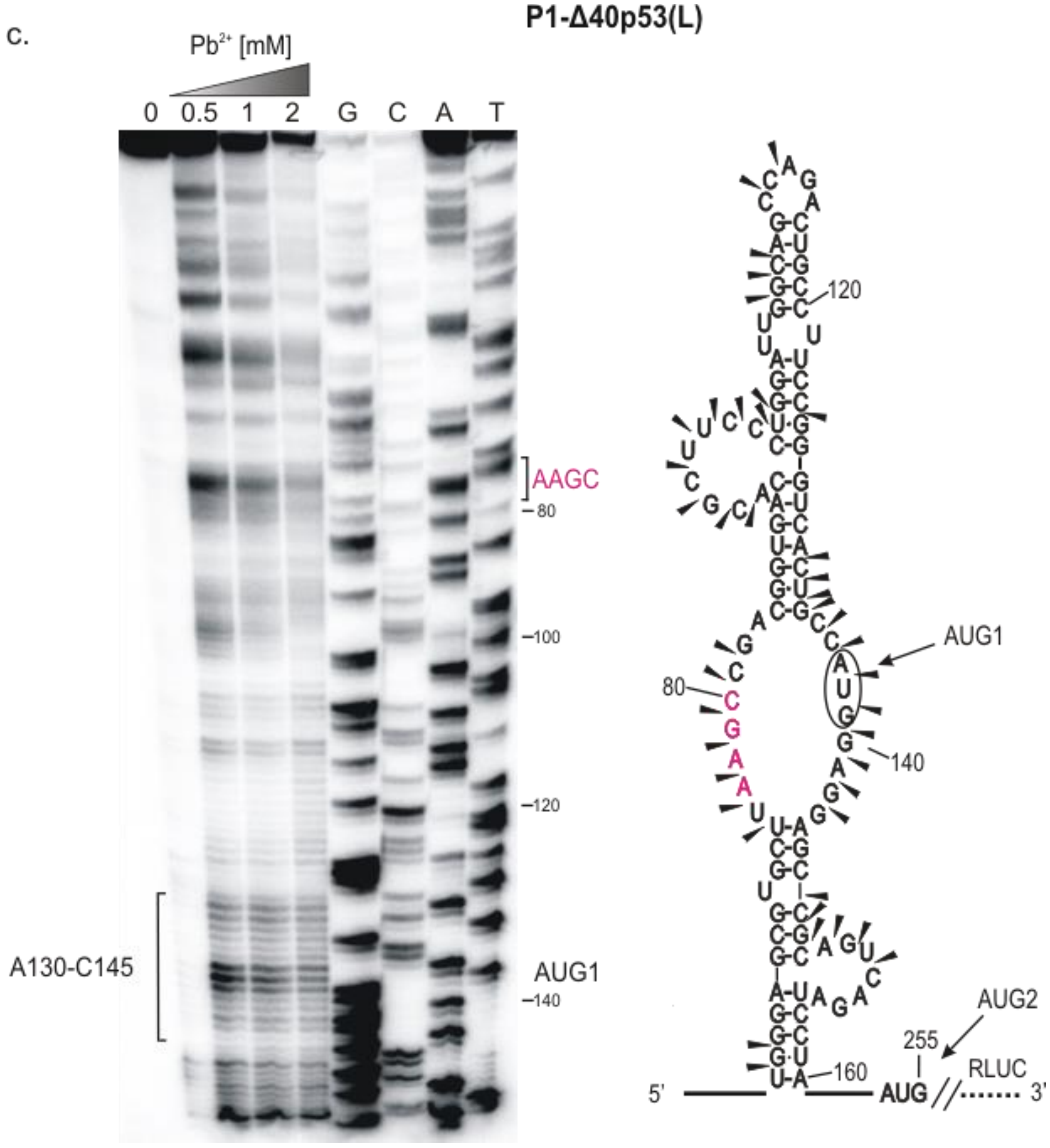

(c) Secondary structure analysis of the P1-Δ40p53(L) construct by Pb^{2+}-induced cleavage mapping. The initiation codon AUG1 is embedded in an internal loop. The introduced mutations are marked in red on the gel and on the secondary structure model. Description of the gel and structure model as in panel a.

d.

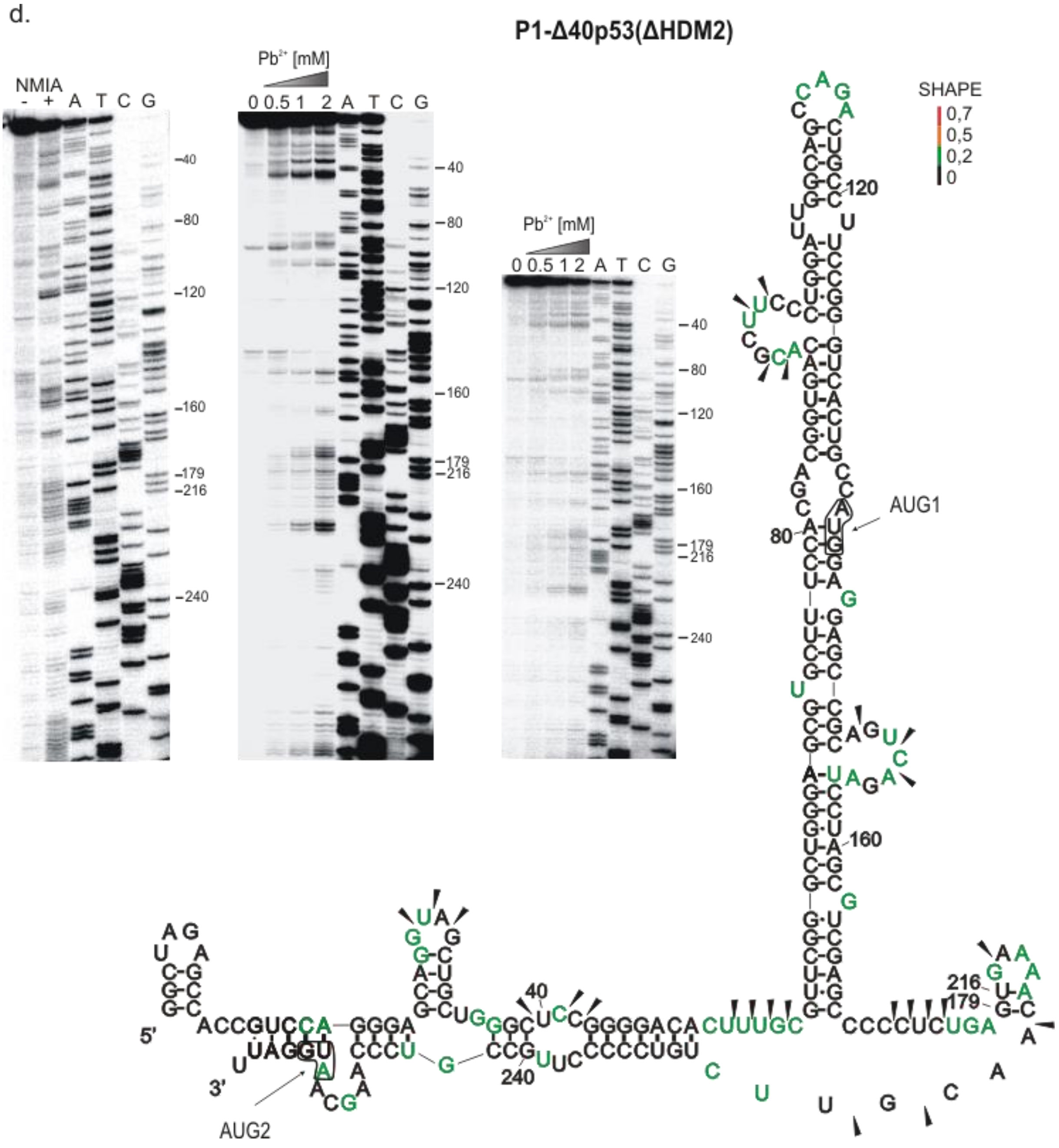

(d) Secondary structure probing of the P1-Δ40p53(ΔHDM2) construct by Pb^{2+}-induced cleavage and SHAPE methods. Lanes '-' and '+' on the gel shown on the left indicate the absence or addition of NMIA reagent, whereas G, C, A and T denote sequencing reactions with guanosine, cytosine, adenine and thymine dideoxy nucleotides, respectively. The description of the gel with Pb^{2+}-induced cleavages as in panel a. A simplified secondary structure model is shown on the right with SHAPE reactivity marked in colours and Pb^{2+}-induced cleavage sites represented by black arrows.

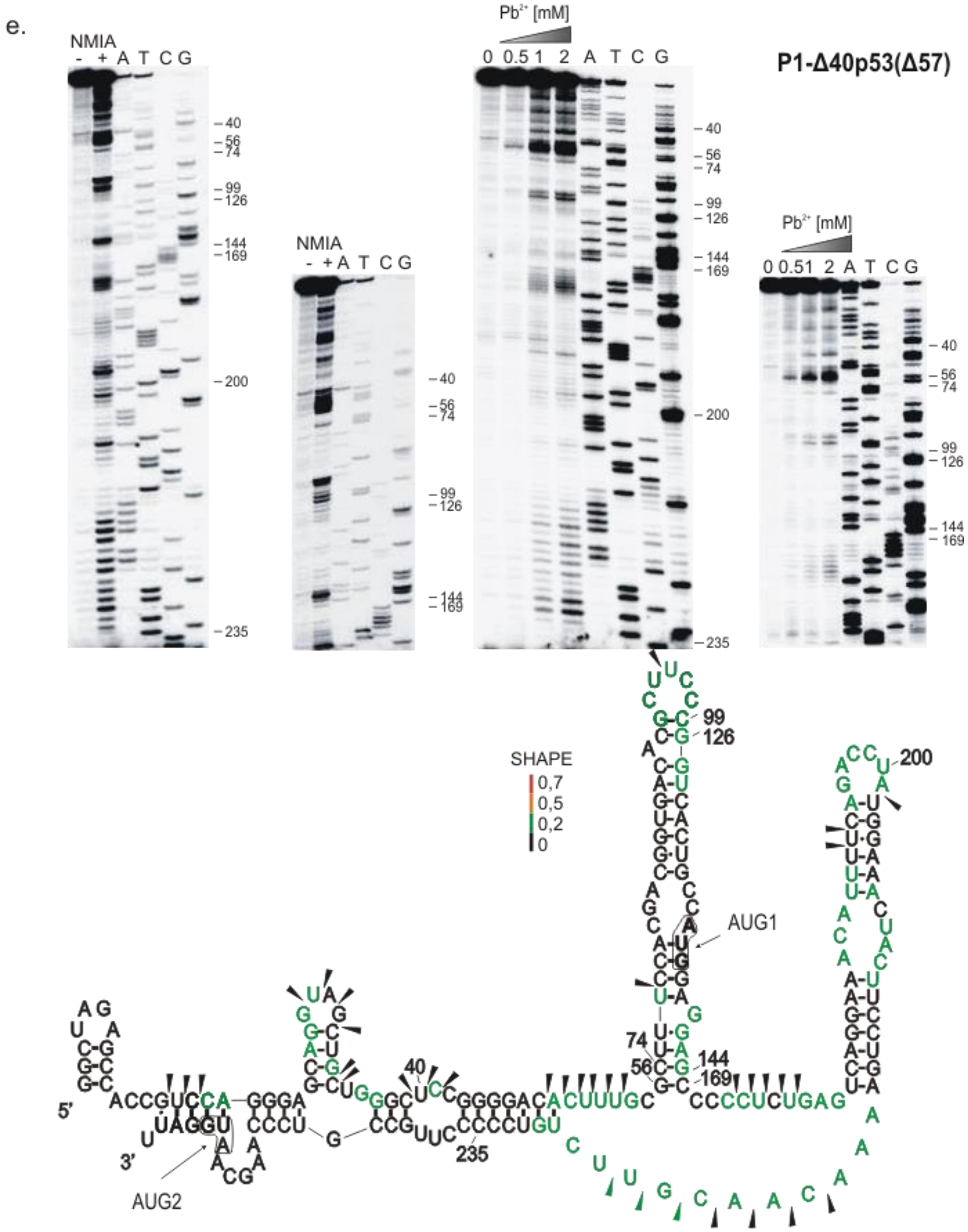

(e) Secondary structure probing of the P1-Δ40p53(Δ57) mRNA construct by Pb^{2+}-induced cleavage and SHAPE methods. The description of the gels and secondary structure model are the same as in panel d.

Supplementary Figure S3: *In vitro* translation and western blot assay of the model mRNA constructs performed in the rabbit reticulocyte lysate (RRL) system. *In vitro* translation was performed in the RRL System (Promega), similarly as described in Materials and Methods. Briefly, the reaction contained 17.5 µl RRL, 20 µM aminoacid mixture minus methionine, 20 µM aminoacid mixture minus leucine, 10 U of RiboLockRNase inhibitor (Thermo Scientific) and 2.5 pmol of capped RNA which was previously denatured for 5 min at 65°C. The final volume of the reaction was 25 µl and it was conducted for 90 min at 30°C. Afterwards, the reaction was treated with 0.16 µg of RNase A for 5 min at 20°C, and denatured for 2 min at 80°C in the presence of SDS Sample Buffer and 100 mM DTT. The reaction products were analysed in 15% SDS-PAGE, followed by western blot analysis. The blot was probed with anti-Renilla Luciferase antibody diluted 1:1000 (Abcam no: ab187338). Subsequently, the primary antibody was detected by goat anti-rabbit-HRP (Thermo Scientific Pierce) and visualized by using the enhanced chemiluminescent visualization (ECL+) system (Thermo Scientific Pierce). The western blot proved that luciferase was synthesized from the applied mRNA constructs.

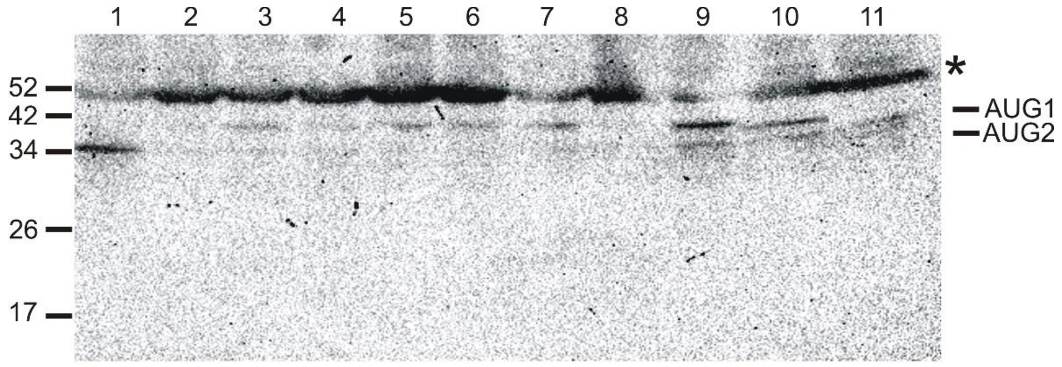

1. P1-p53
2. P0-p53
3. P1-Δ40p53
4. P0-Δ40p53
5. P1-Δ40p53(gUG2)
6. P0-Δ40p53(gUG2)
7. P1-Δ40p53(L)
8. P1-Δ40p53(S)
9. P1-Δ40p53(ΔHDM2)
10. P1-Δ40p53(Δ57)
11. P1-Δ40p53(ΔA224)

*Non-specific bands

Supplementary Figure S4: *In vitro* translation and luciferase reporter gene assay of the model mRNA constructs performed in the rabbit reticulocyte lysate (RRL) system. (a) The graph shows the relative luciferase activity (RLU) of P1-Δ40p53(ΔA224) mRNA and P1-Δ40p53 mRNA construct as a control. P1-Δ40p53(ΔA224) is a derivative of P1-Δ40p53 containing a single adenosine residue deleted from position 224. The mutation was introduced using site-directed mutagenesis of P1-Δ40p53 dsDNA with primers: F224A: 5'-CTTCCTG AAAACACGTTCTGTCC-3' and R224A: 5'-GGACAGAACGTGTTTTCAGGAAG-3'. The *in vitro* translation reactions were conducted in RRL in the same conditions as those described in the Materials and Methods section. Due to the shift in the reading frame caused by the deletion of A224 in P1-Δ40p53(ΔA224), a short peptide is produced from AUG1, while active luciferase is synthesized only from AUG2. The maximal luminescence activity for the P1-Δ40p53(ΔA224) construct is approx. 3-fold lower than for the P1-Δ40p53, as shown on the graph. The full-translation time values calculated from three independent experiments were: 7:27 ± 0:06 and 6:58 ± 0:14 for P1-Δ40p53(ΔA224) and P1-Δ40p53 constructs, respectively. **(b)**The autoradiogram shows the translation products of P1-Δ40p53(ΔA224) and P1-Δ40p53 and mRNA constructs from AUG1 and AUG2 initiation codons. Below the gel, the approximate translation efficiency from AUG1 and AUG2 codons is shown. **(c)** The capped P1-Δ40p53(ΔA224) mRNA construct was translated in RRL in the presence of an increasing concentration of the cap analogue (m7GpppG) to inhibit cap-dependent translation. The amounts of protein product resulting from AUG2 initiation codon were determined, and following quantification and normalization to the values with no cap analogue added, they was displayed on the graph. The graph presents the mean of three independent measurements, with the standard deviations calculated and displayed on the diagram.

a.

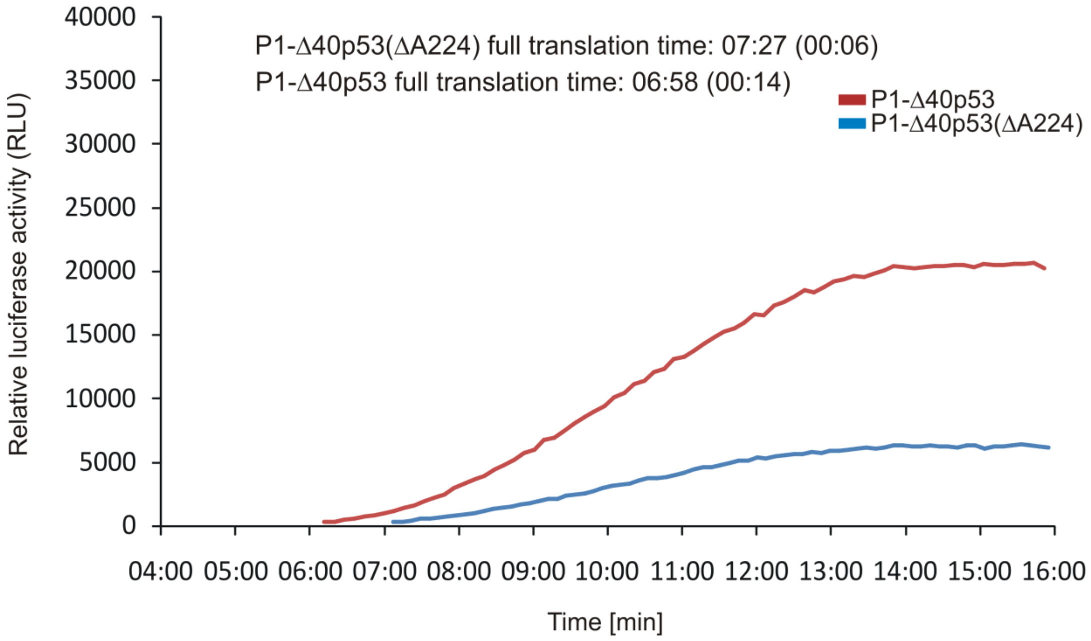

b.

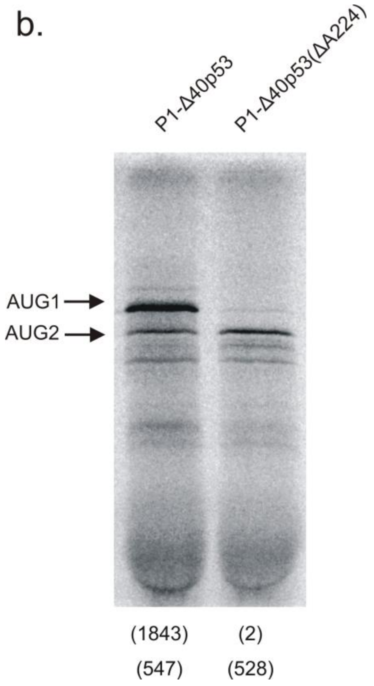
P1-Δ40p53
P1-Δ40p53(ΔA224)
AUG1
AUG2
(1843)
(2)
(547)
(528)

c.

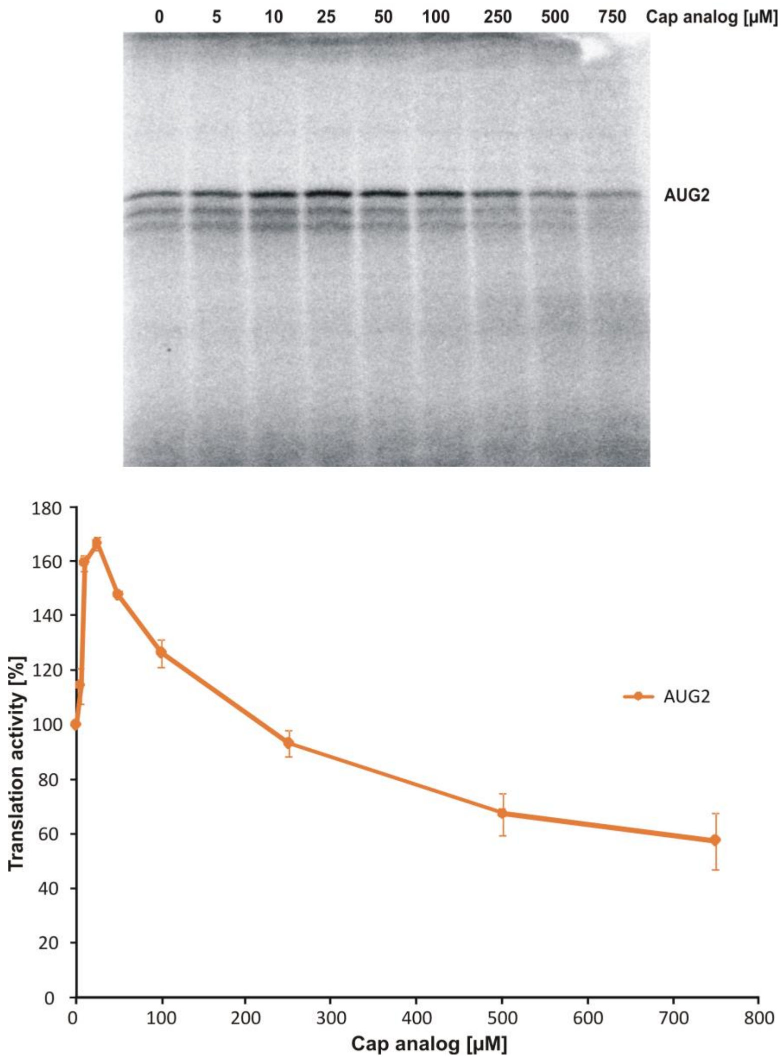
0
5
10
25
50
100
250
500
750
Cap analog [μM]
AUG2
Translation activity [%]
Cap analog [μM]
AUG2
0
20
40
60
80
100
120
140
160
180
0
100
200
300
400
500
600
700
800

Supplementary Figure S5: Full-length images.

Full-length autoradiograms from Figure 2.

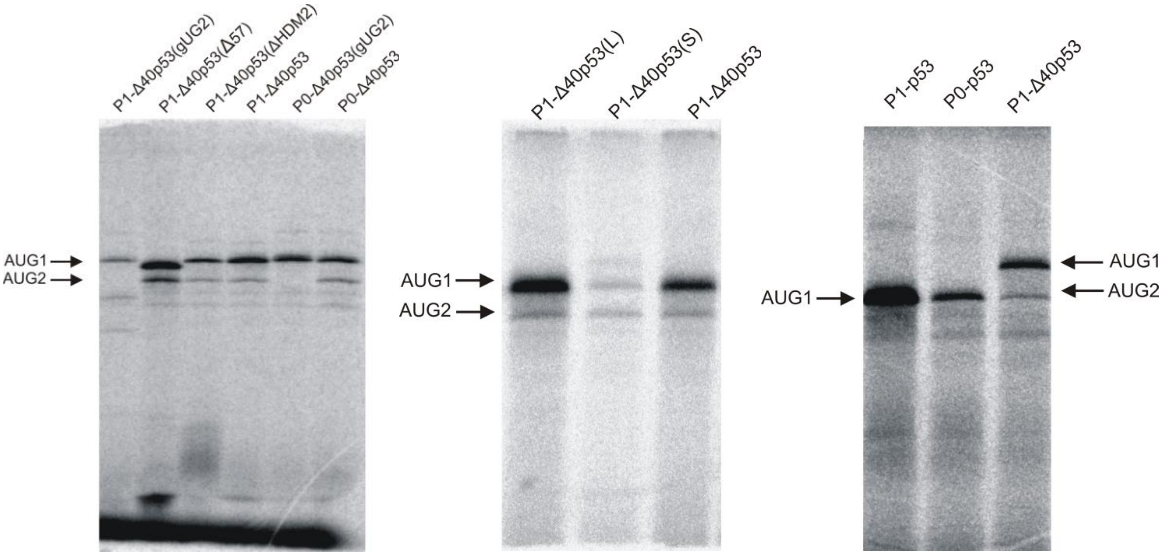

Full-length images of the gels from Figure 6 a), b) and c).

a.

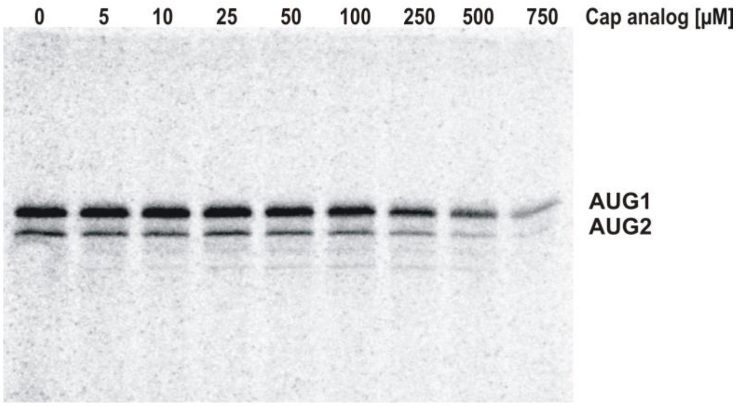

b.

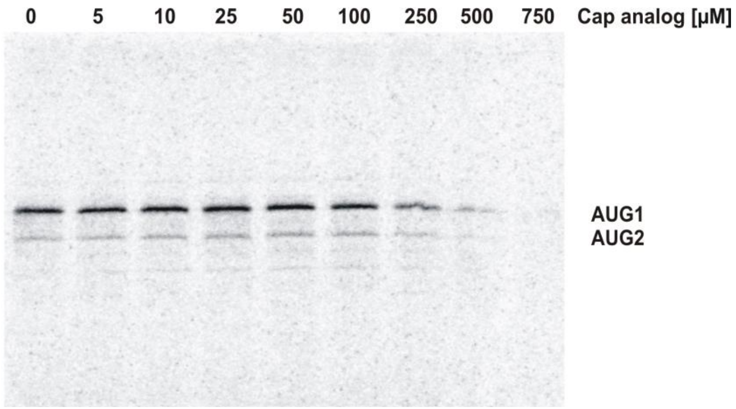
0 5 10 25 50 100 250 500 750 Cap analog [µM]
AUG1
AUG2

c.

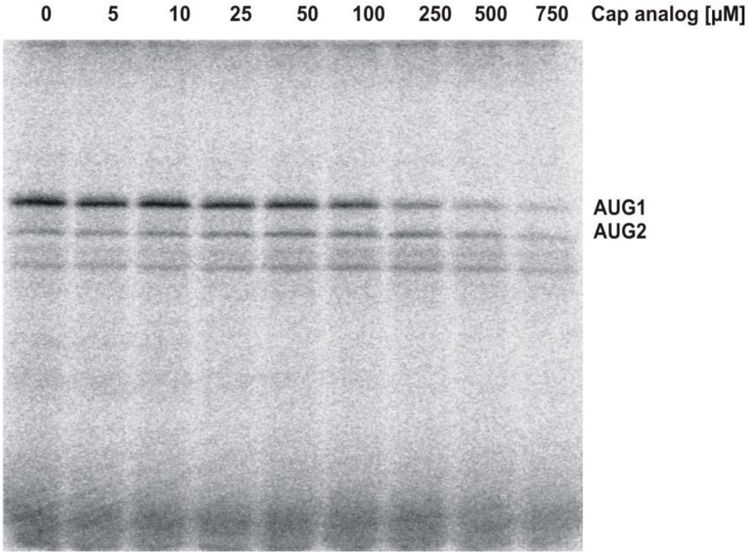
0 5 10 25 50 100 250 500 750 Cap analog [µM]
AUG1
AUG2
